# RTX proteins: a highly diverse family secreted by a common mechanism

**DOI:** 10.1111/j.1574-6976.2010.00231.x

**Published:** 2010-11

**Authors:** Irena Linhartová, Ladislav Bumba, Jiří Mašín, Marek Basler, Radim Osička, Jana Kamanová, Kateřina Procházková, Irena Adkins, Jana Hejnová-Holubová, Lenka Sadílková, Jana Morová, Peter Šebo

**Affiliations:** 1 Institute of Microbiology AS CR v.v.i., Academy of Sciences of the Czech RepublicPrague, Czech Republic; 2 Institute of Biotechnology AS CR v.v.i., Academy of Sciences of the Czech RepublicPrague, Czech Republic

**Keywords:** bacterial toxin, type I secretion system, RTX locus, calcium-binding repeats

## Abstract

Repeats-in-toxin (RTX) exoproteins of Gram-negative bacteria form a steadily growing family of proteins with diverse biological functions. Their common feature is the unique mode of export across the bacterial envelope via the type I secretion system and the characteristic, typically nonapeptide, glycine- and aspartate-rich repeats binding Ca^2+^ ions. In this review, we summarize the current state of knowledge on the organization of *rtx* loci and on the biological and biochemical activities of therein encoded proteins. Applying several types of bioinformatic screens on the steadily growing set of sequenced bacterial genomes, over 1000 RTX family members were detected, with the biological functions of most of them remaining to be characterized. Activities of the so far characterized RTX family members are then discussed and classified according to functional categories, ranging from the historically first characterized pore-forming RTX leukotoxins, through the large multifunctional enzymatic toxins, bacteriocins, nodulation proteins, surface layer proteins, up to secreted hydrolytic enzymes exhibiting metalloprotease or lipase activities of industrial interest.

## Introduction

With molecular cloning and DNA sequencing taking grounds in bacteriology labs, it has become clear since 1987 that a novel family of large secreted cytolytic toxins of Gram-negative pathogens emerged. Summarizing the similarities of the first five characterized determinants in a seminal MicroReview in 1991, Rodney A. Welch first introduced the concept of the RTX family of proteins characterized by the presence of arrays of glycine- and aspartate-rich nonapetide repeats. He predicted that this was a broadly disseminated family, while at that time it was difficult to imagine how broad and variable it could be.

RTX proteins are produced by a variety of Gram-negative bacteria and exhibit two common features. The first is the presence of repetitions of glycine- and aspartate-rich sequences, typically nonapeptides, which are located in the carboxy-terminal portion of the protein. These form numerous sites for the binding of Ca^2+^ ions and are at the origin of the historical name of the protein family, where RTX stands for repeats in toxin (Welch, 2001). The second key feature is the unique mode of secretion of RTX proteins via the type I secretion system (TISS). Protein translocation occurs through an oligomeric secretion channel spanning across the entire Gram-negative bacterial cell envelope (i.e. cytoplasmic membrane, periplasmic space and outer membrane). These dedicated ATP-binding cassette (ABC) transporter-based secretion apparati recognize uncleavable C-terminal secretion signals and mediate a single-step translocation of the RTX polypeptides from bacterial cytosol across both the inner and the outer bacterial membrane, directly into the extracellular space and without a periplasmic secretion intermediate. The *rtx* genes and genes needed for secretion are mostly located within a single larger *rtx* locus.

RTX proteins represent a family of proteins that exhibit a wide range of activities and molecular masses from 40 to >600 kDa. A prominent and historically first described group of RTX proteins consists of toxins, mostly exhibiting a cytotoxic pore-forming activity, often first detected as a hemolytic halo surrounding bacterial colonies grown on blood agar plates (Goebel & Hedgpeth, 1982; Muller *et al.*, 1983; Felmlee *et al.*, 1985; Welch, 1991;).

While the word ‘toxin’ is, for historical reasons, embodied in the name of the RTX protein family, a broad class of RTX proteins also comprises secreted proteases and lipases. These can act as synergistic virulence factors causing tissue damage and/or by eliciting the production of inflammatory mediators. Recently, a subgroup of very large RTX proteins (>3200 amino acid residues) with multiple activities [multifunctional autoprocessing RTX toxins (MARTX)] was discovered. For example, the prototype *Vibrio cholerae* MARTX*_Vc_* was shown to cause rounding of epithelial cells by catalyzing covalent cross-linking of cellular actin. RTX proteins can further act as bacteriocins or contribute to defense against environmental aggression by forming protective bacterial surface (S)-layers. Some RTX proteins were also found to play a role in plant nodulation or *Cyanobacteria* motility, while the biological role of most RTX proteins remains unknown. Bioinformatic mining of the explosively growing database of bacterial genomes indicates that RTX proteins form a large and diverse family of proteins, with a broad spectrum of biological and biochemical activities.

### RTX repeats

The requirement for calcium ions in RTX toxin activities was first documented for *Escherichia coli*α-hemolysin (Short & Kurtz, 1971) and *Bordetella pertussis* CyaA (Hanski & Farfel, 1985). Binding of calcium ions to the repeats of RTX toxins occurs only upon secretion, as the intracellular cytoplasmic calcium concentration in bacteria is quite low (<100 nM) (Gangola & Rosen, 1987). The RTX protein needs to unfold or remains in a floppy conformation before translocation out of the cell through the TISS (Kenny *et al.*, 1991; Koronakis *et al.*, 2000;). Calcium binding to the nonapeptide repeats in the C-terminal portions of these toxins then appears to promote folding and imposes adoption of a functional conformation on the secreted RTX proteins in the extracellular environment (Felmlee & Welch, 1988; Ludwig *et al.*, 1988; Rose *et al.*, 1995; Rhodes *et al.*, 2001;).

**Fig. 1:**
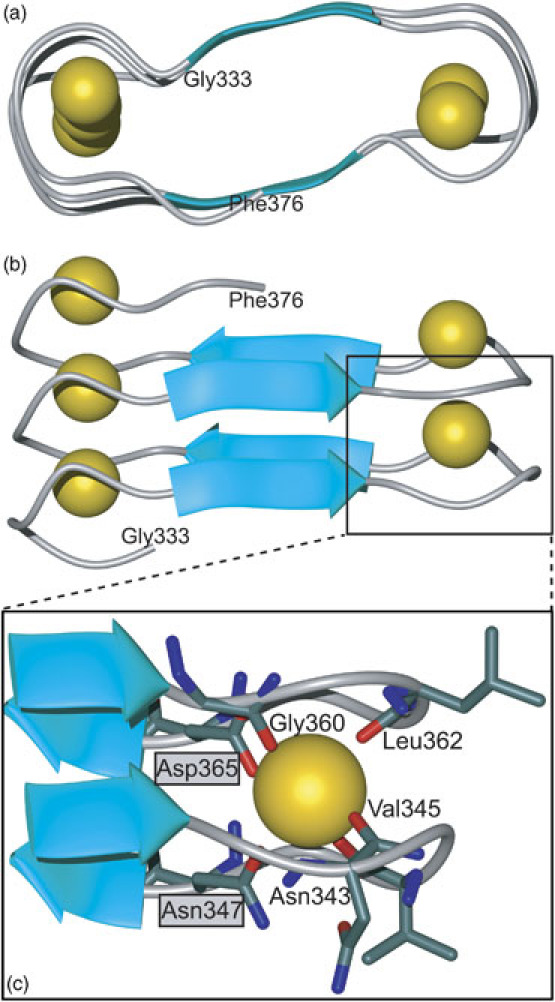
The parallel β-roll structure of the RTX repeats. Top view (a) and side view (b) projection of the RTX repeats comprising residues Gly333 and Phe376 from the three-dimensional model of alkaline protease of *Pseudomonas aeruginosa* (Baumann *et al.*, 1993). The protein backbone, short β-strands and Ca^2+^ ions are represented as grey ribbon, cyan arrows and yellow balls, respectively. (c) A detailed view of the calcium-binding site within the RTX repeats. Each nonapeptide motif forms two half-sites for Ca^2+^ binding, where each Ca^2+^ ion is bound in a six coordinate site between two consecutive turns. The first turn contributes the main chain carbonyls of Asn343 and Val345, and one carboxyl oxygen of Asn347. The second turn contributes the carbonyls of Gly360 and Leu362, as well as the carboxyl oxygen of Asp365. Residues whose side chains directly coordinate the Ca^2+^ ion are highlighted. The carbon atoms of the side chains are green, nitrogens are blue, oxygens are red.

Analysis of the three-dimensional structure of *Pseudomonas aeruginosa* alkaline protease possessing six of the RTX motifs with a consensus sequence X-(L/I/F)-X-G-G-X-G-(N/D)-D, where X means any residue, revealed that the repeated sequences constitute a new type of calcium-binding structure (Fig. 1), called a parallel β-helix or a parallel β-roll (Baumann *et al.*, 1993). In this structure, the first six residues of each motif form a turn that binds calcium, and the remaining three residues build a short β-strand. The arrangement of consecutive turns and β-strands builds up a right-handed helix of parallel β-strands, where one turn of this helix consists in two consecutive nonapeptides. Calcium is then bound within two consecutive turns by the conserved aspartic acids.

The numbers of RTX repeats vary among RTX proteins from <10 to >40. While RTX proteases and lipases typically have a single block of seven to eight RTX nonapeptide repeats, very extensive RTX repeat domains were found recently in the large MARTX or in putative RTX proteins encoded in some sequenced genomes. Somewhere in the middle between the extremes is the RTX domain of CyaA from *B. pertussis*. For example, this is organized in five successive blocks, containing about eight nonapeptide RTX motifs each, which are separated by linkers of variable lengths (Glaser *et al.*, 1988a; Osicka *et al.*, 2000;). The repeat domain of CyaA was then shown to harbor a small number (three to five) of high-affinity (*K*_d_<1 nM) and about 40 low-affinity (*K*_d_∼0.5–0.8 mM) binding sites for Ca^2+^ ions (Rose *et al.*, 1995; Rhodes *et al.*, 2001;).

### TISS

**Fig. 2:**
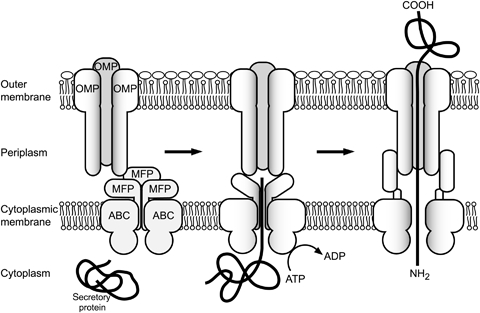
The schematic depiction of the TISS assembly operation. Upon recognition of a C-terminal secretion signal on a given RTX protein translocation substrate, the inner membrane complex formed by an energy-providing ABC transporter and a MFP contacts the trimeric OMP. A sealed channel–tunnel assembly spanning across the entire Gram-negative bacterial cell envelope is formed, through which the RTX protein is exported in a single step from the bacterial cytoplasm directly to the external bacterial surface, without transiting through the periplasmic space. While concentrations of Ca^2+^ ions are typically <100 nM in bacterial cytoplasm, allowing for maintenance of an unfolded RTX domain, millimolar calcium concentrations are typically encountered in host extracellular space colonized by pathogenic bacteria. Loading of RTX repeats of the secreted protein by Ca^2+^ ions then promotes its folding and acquisition of biological activity.

Gram-negative bacteria have evolved several (I–VI) pathways for protein secretion beyond the outer membrane to the extracellular environment (for reviews, see Saier *et al.*, 2008; Fronzes *et al.*, 2009;). The RTX proteins contain an ∼60-residue-long C-terminal secretion signal that is not processed during secretion (Gentschev *et al.*, 1990; Stanley *et al.*, 1991; Sebo & Ladant, 1993;). This is recognized by the *sec*-independent TISS, which mediates the translocation of proteins directly from the cytoplasmic compartment into the extracellular space through a channel spanning the entire cell envelope (Fig. 2).

Type I secretion across the cell wall depends on three specific proteins: a polytopic inner membrane protein with a cytoplasmic ATPase domain operating as an ABC exporter, a membrane fusion protein (MFP) and an outer membrane protein (OMP). The MFP spans out from the inner membrane into the periplasm and contacts both the inner membrane ABC exporter and the OMP. The paradigm of the type I secretion pathway is based on the analysis of the mechanism of secretion of the *E. coli*α-hemolysin (HlyA). The Hly exporter was also shown to promote to some extent the secretion of a number of heterologous RTX proteins expressed in *E. coli*, including the CyaA of *B. pertussis* (Sebo & Ladant, 1993), LtkA of *Aggregatibacter* (formerly *Actinobacillus, Haemophilus*) *actinomycetemcomitans* (Lally *et al.*, 1989), PaxA of *Pasteurella aerogenes* (Kuhnert *et al.*, 2000) or FrpA of *Neisseria meningitidis* (Thompson & Sparling, 1993).

**Fig. 3:**
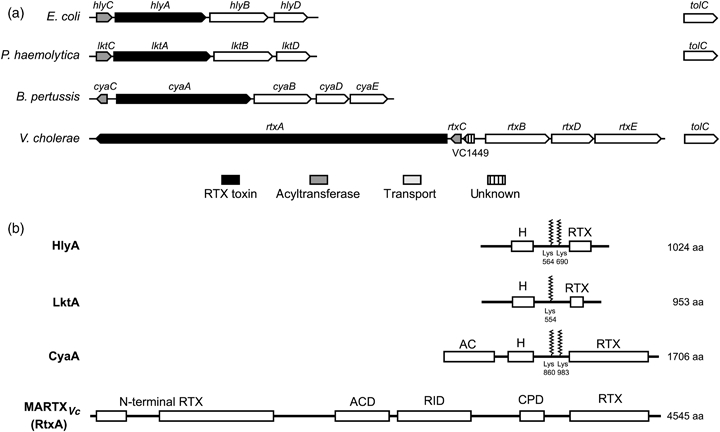
(a) The schematic representation of the *rtx* gene clusters of *Escherichia coli, Pasteurella haemolytica, Bordetella pertussis* and *Vibrio cholerae*. The arrows represent coding regions and transcriptional directions of the *rtx* genes deposited under the following GenBank accession numbers: *E. coli* (NC 000913); *P. haemolytica* PHL213 (NZ AASA00000000); *B. pertussis* Tohama I (NC 002929) and *V. cholerae* N16961 (NC 002505). (b) Domain structures of the RTX cytotoxins HlyA (*E. coli*), LktA (*P. haemolytica*), CyaA (*B. pertussis*) and MARTX_*Vc*_ (*V. cholerae*) with indication of sites of post-translational modification of internal lysines by covalent attachment of fatty acyl residues. The functional domain labelling is as follows: H, haemolytic domain; RTX, RTX domain; AC, adenylate cyclase domain; ACD, actin cross-linking domain; RID, Rho inactivation domain; CPD, cystein protease domain.

The *hlyCABD* operon (Fig. 3a) codes for the toxin activation protein (HlyC), the hemolysin itself (HlyA), the ABC transporter (HlyB) and the MFP protein (HlyD) (Wagner *et al.*, 1983). The outer membrane component (TolC), a multifunctional protein, is encoded outside of the *hly* operon on *E. coli* chromosome (Wandersman & Delepelaire, 1990) and is part of the *mar*-*sox* regulon (Aono *et al.*, 1998). In some other species, however, the gene for a TolC homologue, such as *cyaE* of *B. pertussis*, is comprised in the *rtx* operon (Glaser *et al.*, 1988b). TolC forms a trimeric export channel in the outer membrane and its presence plays a critical role in type I protein secretion (Delepelaire, 2004; Koronakis *et al.*, 2004;).

The structure of TolC was determined by X-ray crystallography (Koronakis *et al.*, 2000). It has been proposed that the trimeric accessory protein HlyD forms a substrate-specific complex with the inner membrane protein HlyB, which subsequently recognizes the C-terminal signal peptide of HlyA. Upon binding of HlyA, the HlyD trimer interacts with the trimeric TolC protein of the outer membrane, inducing its conformational change and export of HlyA (Andersen *et al.*, 2001). This complex appears to be transient, with the inner membrane complex of HlyB, HlyD and TolC disengaging and reverting to a resting state once the substrate has been transported (Thanabalu *et al.*, 1998). The energy necessary for the secretion process depends not only on ATP hydrolysis mediated by HlyB but also on the proton motive force on the inner membrane (Koronakis *et al.*, 1991, 1993, 1995).

However, the work on secretion of *Serratia marcescens* hemoprotein HasA and *Erwinia chrysanthemi* metalloproteases B and C indicated a slightly different order of events (Letoffe *et al.*, 1996), where the ABC transporter and MFP would associate only upon substrate binding.

It is generally assumed that type I secretion involves the translocation of unfolded proteins (Young & Holland, 1999). Secretion of the HasA protein of *S. marcescens* was, indeed, shown to depend on the binding of the chaperone SecB. Moreover, HasA cannot be transported if allowed to fold in the cytoplasm (Delepelaire & Wandersman, 1998; Wolff *et al.*, 2003;). Intriguingly, contact with HlyD was suggested to affect directly or indirectly the folding of HlyA following or during its transit through the translocator (Pimenta *et al.*, 2005).

An atypical TISS has been described for the large MARTX proteins in *Vibrio*. Here, the apparati consists of four proteins, an analogue of HlyB (RtxB), an analogue of HlyD (RtxD), a TolC-like protein and an additional ATP-binding protein RtxE, where both ABC exporter proteins, RtxB and RtxE, appear to be necessary for MARTX protein secretion (Boardman & Satchell, 2004; Lee *et al.*, 2008;).

A gene for a TolC-like OMP is also present in the protease and lipase secretion loci, but is absent from most other operons encoding the RTX toxin family TISS, except for *B. pertussis* (*cya*) or *Moraxella bovis* (*mbx*) loci (Glaser *et al.*, 1988b; Angelos *et al.*, 2003;). A unique genetic organization of TISS for RTX proteins was revealed in *N. meningitidis* (Wooldridge *et al.*, 2005). In contrast to the cistronic organization of the secretion genes for most other *rtx* operons, homologues of *hlyD* and *tolC* genes are flanked by genes normally associated with mobile genetic elements and do not form an operon with the *hlyB* gene. Furthermore, the three genes were shown to be expressed independently and mutation at either locus resulted in the inability to secrete the FrpC and FrpC-like (FrpC2) RTX proteins.

Initially, the gene encoding a TolC homologue had not been recognized in the genomes of *A. actinomycetemcomitans* HK1651 (http://www.genome.ou.edu/act.html) and *Mannheimia haemolytica* PHL213 (Gioia *et al.*, 2006). Crosby & Kachlany (2007), however, found an ORF in *A. actinomycetemcomitans* that encodes the TdeA protein of predicted structural properties similar to TolC and showed that inactivation of the *tdeA* gene resulted in a mutant unable to secrete LtxA.

## Classes of RTX proteins

### RTX cytotoxins

Cytotoxic RTX proteins are produced by a broad range of Gram-negative pathogens (Table 1) including the genera *Escherichia, Bordetella, Proteus, Morganella, Moraxella, Vibrio* and *Kingella*, and members of the *Pasteurellaceae* family (*Mannheimia, Pasteurella* and *Aggregatibacter*). RTX cytotoxins can be roughly divided into two families: the conventional and broadly studied pore-forming leukotoxins and the more recently discovered very large MARTX. The best-studied prototype of the MARTX group is VcRtxA (or MARTX_*Vc*_) from *V. cholerae*, an over 4500-amino-acid-residue-long protein, which causes depolymerization of F-actin stress fibers in a broad range of cell types (Fullner & Mekalanos, 2000).

**Table 1:** Characterized members of the RTX toxin family

RTX toxin	Bacterium	*rtxA* geneproduct size(kDa)	Cell type cytotoxicityand host specificity	Operon structure*	References
HlyA	Uropathogenic *Escherichia coli*	110	Broad	>*CABD*/*tolC*	Goebel & Hedgpeth (1982)
EhxA	Enterohemorrhagic *Escherichia coli*	107	Human and bovine erythrocytes, leukocytes	>*CABD*/*tolC*	Schmidt *et al.* (1995)
CyaA	*Bordetella pertussis*	177	Primarily human CD11b^+^ myeloid phagocytes, activity detectable on all cell types	*C*</>*ABDE*†	Glaser *et al.* (1988a)
LktA	*Mannheimia haemolytica*	102	Bovine leukocytes and platelets, weak hemolytic activity	>*CABD*/*tolC*	Lo *et al.* (1987)
PlLktA	*Mannheimia varigena*	102	Porcine leukocytes	>*CABD*/*tolC*	Chang *et al.* (1993)
PaxA	*Pasteurella aerogenes*	107.5	Cohemolytic activity‡	>*CABD*/*tolC*	Kuhnert *et al.* (2000)
PvxA	*Proteus vulgaris*	110	Erythrocytes	>*CABD*/*tolC*	Welch (1987)
MmxA	*Morganella morganii*	110	Erythrocytes	>*CABD*/*tolC*	Koronakis *et al.* (1987)
LtxA	*Aggregatibacter actinomycetemcomitans*	114	Human and primate leukocytes	>*CABD*/*tolC*	Lally *et al.* (1989)
ApxIA	*Actinobacillus pleuropneumoniae*	110	Broad	>*CABD*/*tolC*	Frey *et al.* (1991)
ApxIIA	*Actinobacillus pleuropneumoniae*	102.5	Narrow against porcine leukocytes, weak hemolytic activity	>*CA*/*tolC*§	Chang *et al.* (1989)
ApxIIIA	*Actinobacillus pleuropneumoniae*	120	Porcine leukocytes, cohemolytic activity‡	>*CABD*/*tolC*	Jansen *et al.* (1993)
ApxIVA	*Actinobacillus pleuropneumoniae*	200	Weak hemolytic activity	>ORF1/*IVA*¶	Schaller *et al.* (1999)
ApxI	*Actinobacillus suis*	110	Horse and porcine lymphocytes, erythrocytes	>*CABD*/*tolC*	Schaller *et al.* (2000)
ApxII	*Actinobacillus suis*	102.5	Horse and porcine lymphocytes, erythrocytes	>*CA*/*tolC*	Burrows & Lo (1992)
ApxII	*Actinobacillus porcitonsillarum*	102.5	Lymphocytes, erythrocytes	>*CABD*/*tolC*	Kuhnert *et al.* (2005)
AqxA	*Actinobacillus equuli*	110	Horse and porcine lymphocytes, erythrocytes	>*CABD*/*tolC*	Berthoud *et al.* (2002)
VcRtxA‖	*Vibrio cholerae*	484	Monkey kidney fibroblasts, human laryngeal epithelial cells**	*ACchp*<>*BDE*/*tolC*††	Lin *et al.* (1999)
VvRtxA‡‡	*Vibrio vulnificus*	550	Human intestinal epithelial cells	*ACchp*<>*BDE*/*tolC*††	Chen *et al.* (2003)
MbxA	*Moraxella bovis*	99	Bovine erythrocytes, leukocytes	>*CABD*/*tolC*	Angelos *et al.* (2003)
RTX cytotoxin	*Kingella kingae*	?	Respiratory epithelial, synovial, macrophage-like cells	>*CABD*/*tolC*	Kehl-Fie & St Geme (2007)

* *A*-structural gene, *BDE*/*tolC-*components of the type I secretion apparatus, *C*-acyltransferase. With the exception of the *cyaA* and *mbxA* gene clusters, the *CABD*/*tolC* operon structure is in a 5′–3′ gene order of *CABD* with *tolC* unlinked and encoded at a distant locus. The transcriptional organization of the *RTX* operons is indicated by arrowheads.

† *cyaE* is homologous to *tolC* from *Escherichia coli*.

‡ The CAMP test for cohemolytic activity was performed on blood agar plates using a β-hemolytic *Staphylococcus aureus* strain (Christie *et al.*, 1944).

§ Not in an operon with type I secretion genes B and D. The secretion of ApxIIA is mediated by ApxIB and ApxID.

¶ ORF1 appears to be required for ApxIV activity. Nothing is known about the secretion of ApxIV.

∥ The *rtxA*-like genes from insect pathogens *Photorhabdus luminescens* and *Xenorhabdus bovienii* (Duchaud *et al.*, 2003; Venter *et al.*, 2004;) were identified by genome sequence analysis.

** No cytolytic activity, causes depolymerization of F-actin by cross-linking of G-actin.

†† The ORF named *chp* encodes a conserved hypothetical protein; the *rtxE* gene encodes additional ATPase that is related to *rtxB*.

‡‡ RtxA toxin from *Vibrio vulnificus* lacks the actin cross-linking domain (Sheahan *et al.*, 2004).?, Product size unknown.

### Pore-forming RTX cytotoxins

Pore-forming RTX cytotoxins represent a unique class of bacterial proteins that share (1) the requirement for post-translational activation through amide-linked fatty acylation of internal lysine residues; (2) possess a hydrophobic domain that was shown or is presumed to form cation-selective pores in target cell membranes; (3) are exported by TISSs; and (4) upon secretion are activated for exerting biological activity by binding calcium ions within the acidic glycine- and aspartate-rich nonapeptide repeats.

Based on the most obvious and/or historically first characterized activity, RTX toxins were divided into hemolysins and leukotoxins. The so-called RTX ‘hemolysins’ were initially found to exhibit a limited target cell and species specificity, while the activity of leukotoxins was considered to be species and cell-type specific (Welch, 1991; Coote, 1992;). For example, *E. coli*α-hemolysin (HlyA) appears to be rather promiscuous, exhibiting a well-detectable cytotoxic activity on a wide spectrum of cells from various species, including erythrocytes, granulocytes, monocytes, endothelial cells or renal epithelial cells from mice, ruminants and primates (Gadeberg & Orskov, 1984; Keane *et al.*, 1987; Bhakdi *et al.*, 1989, 1990; Mobley *et al.*, 1990; Suttorp *et al.*, 1990; Crosby & Kachlany, 2007). In turn, the leukotoxins of *A. actinomycetemcomitans* (LtxA) and *M. haemolytica* (LktA) appear to be quite selective and cytotoxic only to a restricted group of cell types in a species-specific manner (Shewen & Wilkie, 1982; Taichman *et al.*, 1984, 1987; Strathdee & Lo, 1989).

However, this traditional classification as ‘hemolysins’ (pore-forming cytolysins) and leukotoxins appears to be somewhat obsolete. Even the ‘promiscuous’α-hemolysin of *E. coli* (HlyA) and the ‘hemolytic’ adenylate cyclase (AC) toxin (CyaA) of *Bordetella* were now found to preferentially bind and target leukocytes expressing the β_2_-integrins LFA-1 and Mac-1, respectively (Lally *et al.*, 1997; Guermonprez *et al.*, 2001;). It appears more appropriate to assume that all pore-forming RTX toxins are primarily leukotoxins, with selectivity for leukocytes being, at least for some of them, eroded at supraphysiological toxin concentrations. The residual activity of the most potent cytolytic (pore-forming) leukotoxins, with a less narrow host spectrum, would then be readily detected as cytolytic activity towards a broader variety of cell types, including erythrocytes. In contrast to these ‘hemolysins,’ the true ‘leukotoxins’ would lack any obvious activity on other cell types other than leukocytes from a certain host species.

#### Post-translational activation of RTX cytotoxins by covalent fatty acylation

The cytolytic (pore-forming) RTX leukotoxins are synthesized as inactive protoxins that undergo post-translational activation before export from the toxin-producing bacteria. This consists in post-translational modification of ɛ-amino groups of internal lysine residues within conserved acylation sites by covalent attachment of amide-linked fatty acyl residues (Fig. 3b). This reaction is catalyzed by the RtxC acyltransferases expressed together with the protoxins (Goebel & Hedgpeth, 1982; Barry *et al.*, 1991; Sebo *et al.*, 1991;). The mechanism of this novel type of protein acylation was analyzed in substantial detail for the prototype RTX toxin-activating and acyl-ACP-dependent protein acyltransferase HlyC, which converts the *E. coli* proHlyA to mature α-hemolysin toxin HlyA (Issartel *et al.*, 1991; Stanley *et al.*, 1994, 1998; Ludwig *et al.*, 1996). HlyC uses the fatty acyl residues carried by acyl-ACP to form a covalent acyl-HlyC intermediate, which transfers the fatty acyl residues to the ɛ-amino groups of Lys^564^ and Lys^690^ residues of proHlyA (Worsham *et al.*, 2001, 2005). Several residues, including Ser^20^ and His^23^, were identified as being potentially involved in the catalysis of acyl transfer by HlyC (Issartel *et al.*, 1991; Trent *et al.*, 1998, 1999a, b, c). Acyl-ACPs carrying various fatty acyl residues, including the palmitic (C16:0) and palmitoleic (C16:1) residues most common in *E. coli*, could be efficiently used *in vitro* as acyl donors for modification of HlyA, while acyl-CoA is not used as a substrate by HlyC (Issartel *et al.*, 1991; Trent *et al.*, 1998;). *In vivo*, however, HlyC exhibits a high selectivity for C14:0 myristic acid, which was found to constitute about 68% of the acyl chains covalently linked to Lys^564^ and Lys^690^ of native HlyA (Lim *et al.*, 2000). Surprisingly, the extremely rare odd carbon-saturated C15:0 and C17:0 fatty-acyl residues were found to constitute the rest of the *in vivo* acylation of HlyA from two different clinical *E. coli* isolates (Lim *et al.*, 2000). The biological relevance of the use of odd-carbon acyl residues for activation of HlyA as well as the mechanism by which HlyC selects these acyl-ACP loaded by the extremely rare acyl residues remains to be clarified.

The role of acylation in toxin activity was analyzed for *B. pertussis* CyaA, where the extent of fatty acylation *in vivo* was found to depend on the producing strain. Initially, the *Bp*-CyaA extracted from a Tohama I-type *B. pertussis* 338 was found to be monoacylated by a single palmitoylation at the Lys^983^ residue only (Hackett *et al.*, 1994). Further work confirmed that the acylation of Lys^983^ was necessary and sufficient for the activation of CyaA (Basar *et al.*, 2001; Masin *et al.*, 2005;). The CyaA sequence, however, comprises two characteristic acylation sites conserved in the RTX cytolysin family, suggesting that CyaA can also be acylated on a second lysine residue, Lys^860^. The recombinant r-*Ec*-CyaA toxin produced in the presence of CyaC in *E. coli* was, indeed, found to bear a second acylation at Lys^860^ (Hackett *et al.*, 1995). Moreover, recombinant r-*Bp*-CyaA protein overproduced by a *B. pertussis* 18323/pBN strain was also later found to be acylated on both Lys^860^ and Lys^983^ residues (Havlicek *et al.*, 2001). The reduced specific hemolytic activity of r-*Ec*-CyaA was then attributed to the modification by mainly the unsaturated palmitoleic (*cis*Δ9 C16:1) fatty-acyl groups when produced in *E. coli*, while exclusively saturated C16:0 palmityl residues were found attached to *Bp*-CyaA in *B. pertussis* (Havlicek *et al.*, 2001). Furthermore, acylation of each of the Lys^860^ or Lys^983^ residues alone was necessary and sufficient for conferring CyaA a full capacity to tightly bind its α_M_β_2_ integrin receptor (CD11b/CD18). The mutant CyaA-K983R, acylated only on Lys^860^, still exhibited a fairly high (∼20%) cytotoxic activity towards murine monocytic cells expressing CD11b/CD18, when compared with CyaA-K860R mutant monoacylated on the Lys^983^ residue alone, or to intact r-*Ec*-CyaA acylated on both Lys^860^ and Lys^983^ residues (Masin *et al.*, 2005). Acylation of Lys^983^ appears, in turn, to be absolutely essential for the residual cytolytic activity of CyaA on cells lacking CD11b/CD18 (Basar *et al.*, 1999, 2001).

Pore-forming RTX toxins require fatty acylation for all known cytotoxic activities. However, the exact role of the post-translational modification in the mechanism of action is not truly understood. The nonacylated proHlyA and proCyaA form pores in planar lipid bilayers with a much reduced propensity, but the formed pores have quite similar properties as the pores generated by acylated toxin (Ludwig *et al.*, 1996; Masin *et al.*, 2005;). Both nonacylated proHlyA and proCyaA are also quite active in penetrating a naked liposome membrane (Soloaga *et al.*, 1996; Masin *et al.*, 2004;), suggesting that the acyl residues are not essential for toxin penetration into the membrane lipid bilayer. Recent evidence indicated that fatty-acylation status and nature may modulate toxin oligomerization and is essential for productive binding of RTX toxins to target cell receptors, allowing the cytotoxic action to occur (Sun *et al.*, 1999; El-Azami-El-Idrissi *et al.*, 2003; Thumbikat *et al.*, 2003; Masin *et al.*, 2005;).

#### Pore-forming activity and interaction with the cell membrane in the absence of a specific cell receptor

The highly potent and less specific cytolytic RTX leukotoxins, such as *E. coli* HlyA or *Bordetella* CyaA, also exhibit a readily detectable activity on cells other than leukocytes. Their interaction with the target cell membrane devoid of a specific proteinaceous receptor appears to occur in two steps, starting with a reversible adsorption of the toxin that is sensitive to electrostatic forces, which is then followed by an irreversible membrane insertion (Bakas *et al.*, 1996; Ostolaza *et al.*, 1997;). Adsorption of RTX toxins is detectable on both toxin-sensitive cells and on certain toxin-resistant cells (Iwase *et al.*, 1990). Studies with the isolated calcium-binding domain of HlyA revealed that this part of the protein may adsorb on the membrane in the early stages of HlyA–membrane interaction (Sanchez-Magraner *et al.*, 2007). Recent results with CyaA, HlyA and LtxA showed that these toxins exhibit a weak lectin activity and recognize and bind the N-linked oligosaccharides of their β_2_ integrin receptors (Morova *et al.*, 2008). This raises the possibility that the initial unsaturable binding of RTX cytotoxins to various cells might occur through the recognition of glycosylated membrane components, such as glycoproteins and gangliosides.

Whether proteinaceous receptors are involved in binding of RTX toxins to cell types other than leukocytes remains an open question. For example, earlier dose–response binding assays indicated an upper limit of 4000 HlyA binding sites per erythrocyte, implying at least some degree of specificity (Bauer & Welch, 1996b). Eberspächer *et al.* (1989), however, did not observe any saturability of binding of HlyA to erythrocytes, suggesting that binding was receptor independent. These data are compatible with later observations of Cortajarena *et al.* (2001, 2003), showing that HlyA can use the abundant glycophorin protein as a high-affinity receptor on erythrocytes. HlyA binding and action on erythrocytes was blocked by antibodies binding glycophorin, by a competing peptide comprising residues 914–936 of HlyA or upon glycophorin digestion with trypsin.

Once HlyA has inserted into the cell membrane, it appears to undergo an irreversible conformational change (Moayeri & Welch, 1997), after which it cannot be recovered from the membrane without the use of detergents (Bhakdi *et al.*, 1986). However, the mechanism of membrane insertion and pore formation by RTX toxins remains poorly understood. Several studies confirmed that the hydrophobic domain of the N-terminal half of the pore-forming RTX leukotoxins is critical for their ability to form transmembrane pores (Ludwig *et al.*, 1987; Glaser *et al.*, 1988a; Cruz *et al.*, 1990; Osickova *et al.*, 1999; Basler *et al.*, 2007;). Other studies also showed that the hydrophobic region of *E. coli* HlyA was responsible for the insertion of the toxin into the target membrane (Hyland *et al.*, 2001; Schindel *et al.*, 2001;). Biophysical studies demonstrated that RTX toxins form cation-selective pores of a defined size and with short lifetimes of only a few seconds (Menestrina *et al.*, 1987, 1996; Benz *et al.*, 1989, 1994; Szabo *et al.*, 1994; Lear *et al.*, 1995; Maier *et al.*, 1996; Schmidt *et al.*, 1996; Karakelian *et al.*, 1998). At higher toxin concentrations, however, these pores may change subunit stoichiometry over time and aggregate into larger lesions in the cell membrane (Moayeri & Welch, 1994).

Whether pore formation by the RTX leukotoxins depends on the toxin oligomerization step remained a matter of controversy. HlyA has been recovered from target membranes only as a monomer (Menestrina *et al.*, 1987; Eberspächer *et al.*, 1989; Stanley *et al.*, 1993;). On the other hand, the dose–response analyses indicated that the lytic activity on target cells was a highly cooperative function of toxin concentration, suggesting that oligomerization was involved in RTX toxin pore formation (Cavalieri & Snyder, 1982; Simpson *et al.*, 1988; Bhakdi *et al.*, 1989; Taichman *et al.*, 1991b; Betsou *et al.*, 1993; Szabo *et al.*, 1994; Bauer & Welch, 1996a; Gray *et al.*, 1998; Osickova *et al.*, 1999;). Moreover, *in vitro* complementation within pairs of individually inactive deletion variants of *E. coli* HlyA or *B. pertussis* CyaA allowed to restore, at least in part, the hemolytic and cytotoxic activities, suggesting that two or more toxin molecules had aggregated to form a pore. This substantiated the view that oligomerization was involved in pore formation by HlyA or CyaA (Ludwig *et al.*, 1993; Iwaki *et al.*, 1995; Bejerano *et al.*, 1999;). Recent results obtained with CyaA revealed the presence of rather unstable (dynamic stoichiometry) CyaA oligomers in the erythrocyte membrane, revealing a correlation between oligomerization of CyaA mutants in the membrane and their pore-forming capacity (Vojtova-Vodolanova *et al.*, 2009).

#### Binding through cell-specific receptors

Recently, it has become increasingly clear that even the more ‘promiscuous’ RTX leukotoxins bind leukocytes through specific proteinaceous receptors of the β_2_ integrin family. For example, CyaA of *B. pertussis* was shown to use the α_M_β_2_ (CD11b/CD18) integrin (known also as a complement receptor 3 or Mac-1) as a target-cell specific receptor (Guermonprez *et al.*, 2001). HlyA of *E. coli* was also found to specifically bind leukocytes (Welch, 1991) and to interact with the β_2_ integrin CD11a/CD18 at low concentrations (Lally *et al.*, 1997). Other studies, however, indicated that the binding of HlyA to cells occurred in a nonsaturable manner and the toxin did not interact with a specific protein receptor on granulocytes (Valeva *et al.*, 2005). These contradictory results await definitive clarification and it is possible that too low concentrations of HlyA were used in the latter study and saturation of the abundant CD11a receptor was not reached. Moreover, *A. actinomycetemcomitans* and *M. haemolytica* leukotoxins (LtxA and LktA, respectively) are specific for human and bovine leukocytes, respectively, and were also found to interact with CD11a/CD18 (Lally *et al.*, 1997; Ambagala *et al.*, 1999; Li *et al.*, 1999;). The initial interaction of RTX leukotoxins with β_2_ integrins then appears to rely on the recognition of N-linked glycans, as revealed for *A. actinomycetemcomitans* LtxA, *E. coli* HlyA and *B. pertussi*s CyaA (Morova *et al.*, 2008).

#### Elevation of calcium concentration in target cells

Elevation and modulation of free cytosolic calcium concentrations are basic strategies of host cell manipulation by pathogens (TranVan Nhieu *et al.*, 2004). Cytosolic calcium levels are tightly controlled and their modulation is part of most prominent cellular signalling pathways that regulate many cellular processes (Berridge *et al.*, 1998, 2000). The initial observation that HlyA of *E. coli* was responsible for significant calcium influx into cells was made by Jorgensen *et al.* (1983). This was corroborated by demonstrating that pore-forming RTX toxins, such as *M. haemolytica* LktA, *A. actinomycetemcomitans* LtxA or *B. pertussis* CyaA, also caused unregulated calcium influx into target cells (Taichman *et al.*, 1991b; Sun *et al.*, 1999; Fiser *et al.*, 2007;). It has even been proposed that cell killing by RTX toxins was due to unregulated calcium influx, which would initiate cytoskeletal destruction and cell lysis (Welch, 2001). This mechanism may underlie the action of *A. actinomycetemcomitans* LtxA that was reported to promote Ca^2+^ changes in T-cells and to initiate a series of events that involve the activation of calpain, talin cleavage, mobilization of β_2_ integrin molecules into membrane lipid rafts and subsequent cell lysis (Fong *et al.*, 2006). Other findings indicate that the calcium influx induced by sublytic doses of RTX toxins accounts for the induction of inflammatory responses (Hsuan *et al.*, 1999; Uhlen *et al.*, 2000; Cudd *et al.*, 2003b;).

There persists, nevertheless, a controversy on the mechanisms by which specifically the RTX family of toxins would promote calcium influx into cells. Uhlen *et al.* (2000) reported that sublytic doses of *E. coli* HlyA stimulated oscillatory calcium responses in renal epithelial cells through the activation of L-type voltage-gated calcium channels and the subsequent response of IP_3_ receptor channels in the endoplasmic reticulum. Moreover, *M. haemolytica* LktA appears to increase the cytoplasmic Ca^2+^ concentration in bovine alveolar macrophages and neutrophils both by the influx of extracellular Ca^2+^ through voltage-gated channels (Ortiz-Carranza & Czuprynski, 1992; Hsuan *et al.*, 1998; Cudd *et al.*, 2003a;) as well as by promoting the release of Ca^2+^ from stores in the endoplasmic reticulum (Cudd *et al.*, 2003a). However, working with different concentrations of HlyA on different cell lines, others have reported that HlyA induces an increase in cytoplasmic [Ca^2+^]_c_ by allowing a passive influx of Ca^2+^ into cells through toxin pores (Valeva *et al.*, 2005; Koschinski *et al.*, 2006;). Moreover, Fiser *et al.* (2007) reported that *B. pertussis* CyaA caused an increase of [Ca^2+^]_c_ in monocytic cells by a mechanism that is independent of its pore-forming activity or of Ca^2+^ release from intracellular stores and depends on membrane translocation of the N-terminal cell-invasive domain polypeptide, but not on its enzymatic activity. The translocating AC domain, as such, appears, indeed, to participate in the formation of a novel and transient Ca^2+^ influx path in the host cell membrane (Fiser *et al.*, 2007).

These seemingly contradictory results may be reconciled in part by the observed positive feedback effect on initial cell membrane permeabilization due to toxin insertion. As shown for HlyA, ATP leakage and Ca^2+^ influx, accompanying membrane insertion of HlyA, induces the activation of purinergic receptors and pannexin channels that are permeable for monovalent cations and Ca^2+^. This further potentiates the influx of extracellular Ca^2+^ and contributes to cell lysis (Skals *et al.*, 2009).

### Broadly cytolytic RTX leukotoxins (hemolysins)

RTX leukotoxins are typically produced by Gram-negative pathogens and commensals of respiratory, gastrointestinal or reproductive tracts or oral cavities of animals and humans. The characterized broadly cytolytic RTX leukotoxins, thus far classified as ‘hemolysins’, are listed in Table 1. These toxins exhibit a hemolytic activity *in vitro* that is revealed by cultivating corresponding bacteria on sheep blood agar plates. *In vivo*, these toxins induce the production of inflammatory mediators or display cytotoxic and cytolytic effects on host immune cells of different species, thus inducing necrosis, apoptosis and inflammation (Czuprynski & Welch, 1995; Welch, 2001; Frey & Kuhnert, 2002;).

#### HlyA of *E. coli*

Among the best-characterized RTX ‘hemolysins’ is HlyA, the single polypeptide (107 kDa) α-hemolysin secreted by uropathogenic as well as many commensal fecal isolates of *E. coli* (Fig. 3). The N-terminal 200 amino acid hydrophobic domain of HlyA is predicted to contain nine amphipathic α-helices (Hyland *et al.*, 2001), while the C-terminal Ca^2+^-binding domain contains 11–17 of the glycine- and aspartate-rich nonapeptide β-strand repeats (depending on the stringency of the criterium for consensus motif conservation). It is assumed that membrane interaction of HlyA occurs mainly through the amphipathic α-helical domain. However, it has been proposed recently that both major domains of HlyA are directly involved in the membrane interaction of HlyA, the Ca^2+^-binding domain being responsible for the early stages of HlyA docking to the target membrane (Sanchez-Magraner *et al.*, 2007). Similarly, Masin *et al.* (2004) showed that both the pore-forming and the acylation domain of CyaA are required for membrane interaction. Cortajarena *et al.* (2001) observed that a short sequence from the C-terminal domain (amino acid 914–936) was the main HlyA segment binding theα-glycophorin on erythrocytes.

A homologous EhxA protein is produced by the enterohemorrhagic *E. coli* O157:H7 from a gene located on a 90 kbp plasmid (pO157). EhxA exhibits 61% identity to HlyA, but displays a more narrow target cell specificity and binds erythrocytes less efficiently (Bauer & Welch, 1996b; Stanley *et al.*, 1998;), exhibiting virtually no activity against human leukocytes (Bauer & Welch, 1996b).

#### MmxA, MbxA and PvxA of *Enterobacteriaceae* and MbxA of *Moraxellaceae*

‘Hemolysins’ homologous to HlyA were identified by Koronakis *et al.* (1987) as MmxA of *Morganella morganii* and PvxA of *Proteus vulgaris*, where MmxA also exhibited cytotoxic activity towards human polymorphonuclear leukocytes (Eberspächer *et al.*, 1990).

The MbxA secreted by *M. bovis* is implicated in the pathogenesis of infectious bovine keratoconjunctivitis (Brown *et al.*, 1998), where it promotes corneal epithelial cell damage (Rogers *et al.*, 1987; Beard & Moore, 1994;). The *mbxCABD* genes form a classical *rtx* operon that is absent from nonhemolytic strains (Angelos *et al.*, 2003) and is part of a mobile genetic element designated as a pathogenicity island of *M. bovis* (Hess & Angelos, 2006).

#### ApxIA, ApxIIA, ApxIIIA, AqxA and PaxA of *Pasteurellaceae*

A large group of homologous cytolytic RTX ‘hemolysins’ is secreted by bacteria of the genus *Pasteurellaceae*. These include ApxIA, ApxIIA, ApxIIIA of *Actinobacillus* sp. (Chang *et al.*, 1989; Rycroft *et al.*, 1991; Frey & Kuhnert, 2002;) and AqxA of *Actinobacillus equuli* (Berthoud *et al.*, 2002) or PaxA of *Pasteurella aerogenes* (Frey & Kuhnert, 2002). In fact, genetic analysis suggests that RTX determinants might have evolved in *Pasteurellaceae* and spread to other Gram-negative bacteria by horizontal gene transfer.

Studies involving *apx* deletion mutants and trans-complementation experiments show that ApxIIA is essential in the pathogenesis of porcine pleuropneumonia and that the combination of ApxIA and ApxIIA, secreted by certain serotypes of *Actinobacillus pleuropneumoniae*, accounts for the severe course of the disease with a fatal outcome (Reimer *et al.*, 1995). The ApxIIIA protein (Rycroft *et al.*, 1991; Frey *et al.*, 1993a,b;) as well as PaxA of *P. aerogenes* (Kuhnert *et al.*, 2000) are nonhemolytic on erythrocytes, but show a significant CAMP reaction, a cohemolytic reaction dependent on the sphingomyelinase from β-hemolytic *Staphylococcus aureus* (Frey *et al.*, 1994; Kuhnert *et al.*, 2000;). ApxIIIA was shown to be highly cytotoxic against macrophages and was previously called the macrophage toxin (Lally *et al.*, 1989; MacDonald & Rycroft, 1992;). The cytotoxic activity of PaxA, also produced by *Pasteurella mairi*, has not yet been analyzed in detail (Frey & Kuhnert, 2002), but all *P. aerogenes* and *P. mairi* isolated from aborted feta or neonatal septicemia of pigs produced PaxA, while strains devoid of the *paxA* gene are isolated as opportunistic pathogens or commensals (Kuhnert *et al.*, 2000). In general, there are little molecular and functional data available on the role in virulence and host or target cell specificity of most of the RTX toxins from animal pathogens.

### Species-specific RTX leukotoxins

The *sensu stricto* RTX leukotoxins appear to be active only on a restricted group of cell types in a species-specific manner (Frey & Kuhnert, 2002; Henderson *et al.*, 2003; Zecchinon *et al.*, 2005;). For example, the biological effect of LktA produced by *M. haemolytica* is largely restricted to ruminant leukocytes and platelets (Kaehler *et al.*, 1980; Shewen & Wilkie, 1982; Strathdee & Lo, 1987; Brown *et al.*, 1997;), whereas *A. actinomycetemcomitans* LtxA only kills lymphocytes and granulocytes from humans, the Great Apes and Old World monkeys (Taichman *et al.*, 1984, 1987). Both LktA and LtxA, however, also exhibit a detectable hemolytic activity on erythrocytes (Shewen & Wilkie, 1982; Taichman *et al.*, 1991a; Balashova *et al.*, 2006;).

LktA plays a role in the pathogenesis of bovine and ovine pneumonic pasteurellosis (Jeyaseelan *et al.*, 2002), whereas *A. actinomycetemcomitans* LtxA is the main virulence factor of localized aggressive periodontitis in humans (Henderson *et al.*, 2003; Fine *et al.*, 2006; Haubek *et al.*, 2006;). These toxins can inhibit the mobility, chemotaxis and respiratory burst of neutrophils, release inflammatory mediators from granulocytes or macrophages, prevent phagocytosis by polymorphonuclear cells and disrupt the phagosome, thus allowing bacterial invasion of the phagocyte. Altogether, these effects strongly reduce the immune response of the host. The *A. actinomycetemcomitans* LtxA was further reported to interact with Cu, Zn superoxide dismutase, which may protect both the bacteria and the LtxA from reactive oxygen species produced by host inflammatory cells (Balashova *et al.*, 2007).

Cytolytic RTX leukotoxins (Table 1) are proteins of a typical molecular mass of 100–120 kDa that possess similar structural and functional domains and are encoded within similar *rtx* operons as the RTX ‘hemolysins’ (Fig. 3) (Chang *et al.*, 1987; Lo *et al.*, 1987; Lally *et al.*, 1989;). LtxA shares 40–50% amino acid homology with the *E. coli* HlyA and *M. haemolytica* LktA. Similarly, both the nonacylated proLktA and proLtxA were in turn able to bind target cells without increasing the intracellular calcium concentration or inducing cytolysis (Sun *et al.*, 1999; Highlander *et al.*, 2000; Thumbikat *et al.*, 2003; Balashova *et al.*, 2009;). The proLktA can be acylated to mature LktA by *E. coli* HlyC, *Bordetella* CyaC and *Actinobacillus* LtxC (Lally *et al.*, 1994; Westrop *et al.*, 1997;). Substitution of Lys-554 to Thr or Cys reduced the lytic activity of LktA against bovine lymphocytes by only 40%, indicating the presence of a second functionally redundant acylation site (Pellett & Welch, 1996). Recently, two internal lysine residues of *A. actinomycetemcomitans* LtxA (Lys-562 and Lys-687) have been identified as targets for covalent fatty acid modification by the *ltxC* gene product (Balashova *et al.*, 2009). Interestingly, LtxA is the only RTX toxin that has a basic pI of 8.9. Also, the RTX domains of both LktA and LtxA are shorter than that of HlyA and contain only seven and eight calcium-binding nonameric repeats, respectively (Lally *et al.*, 1991b; Kuhnert *et al.*, 1997;). Their hydrophobic domain in the N-terminal part was proposed as the region that spans the target cell membrane and deletions in this region impair the cytolytic and pore-forming activities (Cruz *et al.*, 1990). While the region of LtxA required for the recognition of human target cells appears to span the glycine-rich repeats (Lally *et al.*, 1994), the segment determining specificity for ruminant cells has been associated with the N-terminal portion of LktA (Forestier & Welch, 1991; Thumbikat *et al.*, 2003;).

The production of leukotoxins is increased under anaerobic conditions (Spitznagel *et al.*, 1995; Hritz *et al.*, 1996; Kolodrubetz *et al.*, 2003;) and is regulated by environmental cues, such as the availability of iron (Balashova *et al.*, 2006), temperature (Marciel & Highlander, 2001), sugar levels (Inoue *et al.*, 2001) and the concentration of signalling molecules of the quorum-sensing system (Fong *et al.*, 2001).

A difference in the release of LtxA into the culture medium and retention on the cell surface was observed between nonadherent (smooth) and adherent (rough) strains of *A. actinomycetemcomitans* (Tsai *et al.*, 1984; Lally *et al.*, 1991a; Ohta *et al.*, 1991; Berthold *et al.*, 1992; Kachlany *et al.*, 2000;) and LtxA release into the medium could be related to mutations in the *tad* gene involved in the tight nonspecific adherence of *A. actinomycetemcomitans* (Kachlany *et al.*, 2001). Recently, *A. actinomycetemcomitans* LtxA was also found to be released within outer-membrane vesicles (Kato *et al.*, 2002; Diaz *et al.*, 2006;). These are likely to deliver LtxA to host cells (Demuth *et al.*, 2003), similar to the delivery of *E. coli* heat-labile enterotoxin into mammalian cells (Kesty *et al.*, 2004).

The quite narrow host range of leukotoxins appears to be due to cell-specific binding through the β_2_ integrins. The requirement for β_2_ integrins in cytotoxic action was first observed for *M. haemolytica* LktA, which was not cytotoxic to neutrophils isolated from animals suffering from a bovine leukocyte adhesion deficiency. This deficiency is due to a single point mutation (D128G) in the highly conserved extracellular region of the CD18 subunit, which results in reduced membrane expression of the β_2_ integrin LFA-1 (Shuster *et al.*, 1992; Nagahata, 2004;). In fact, the β_2_ integrins share a common β_2_ subunit, CD18, which is combined with either one of the unique α chains, αL (CD11a), αM (CD11b), αX (CD11c) or αD (CD11d). So far, two of these integrins have been found to bind RTX leukotoxins: α_L_β_2_ integrin, known as lymphocyte function-associated antigen 1 (LFA-1, CD11a/CD18), was shown to bind HlyA, LktA and LtxA, and the α_M_β_2_ integrin binds *Bordetella* CyaA, respectively (Lally *et al.*, 1997). Transfection of an LFA-1-expressing construct rendered an otherwise resistant K562 cell line sensitive to killing by LtxA (Lally *et al.*, 1997). Recently, Kieba *et al.* (2007) explained the species selectivity of LtxA by showing that it recognizes the N-terminal 128 amino acid residues of human CD11a that are missing in the murine LFA-1 counterpart. However, the cysteine-rich tandem repeats of the human CD18 subunit were also reported to interact with LtxA (Dileepan *et al.*, 2007a).

The CD18 subunit of bovine β_2_ integrins was, in turn, identified as the receptor for *M. haemolytica* LktA (Ambagala *et al.*, 1999; Li *et al.*, 1999; Dassanayake *et al.*, 2007;). Expression of either bovine LFA-1 or chimeric LFA-1 (bovine CD18/murine CD11a) rendered the LktA-resistant cells susceptible to LktA (Deshpande *et al.*, 2002; Dileepan *et al.*, 2005;), with the magnitude of LktA-induced cytotoxicity correlating with the levels of LFA-1 expression on the target cell surface (Jeyaseelan *et al.*, 2000; Leite *et al.*, 2000;). It remained, however, controversial as to which part of the CD18 subunit LktA binds, as Gopinath *et al.* (2005) mapped it within amino acids 1–291, while Dileepan *et al.* (2005, 2007b) found it between residues 541 and 581 of the extracellular region of bovine CD18. Nevertheless, Shanthalingam & Srikumaran (2009) have recently shown that the LktA-binding site is formed by amino acids 5–17 of CD18, which, surprisingly, comprise most of the amino acids of the signal peptide that remains intact on mature CD18 molecules on the cell surface.

Leukotoxin activities against target cells are highly dose dependent. At sublytic concentrations, leukotoxins induce an increase in [Ca^2+^]_c_ in cells and activate neutrophils and mononuclear cells to undergo oxidative burst and degranulation (Czuprynski *et al.*, 1991; Maheswaran *et al.*, 1992; Stevens & Czuprynski, 1995; Balashova *et al.*, 2009;). This is accompanied by the production of several lipid mediators and proinflammatory cytokines from human macrophages (Johansson *et al.*, 2000; Kelk *et al.*, 2005, 2008). Pulmonary mast cells were shown to release histamine in response to LktA (Adusu *et al.*, 1994) and LktA has also been shown to inhibit bovine lymphocyte blastogenesis induced by concanavalin A and pokeweed mitogen (Majury & Shewen, 1991). *Mannheimia haemolytica* LktA also affects the adhesion of platelets, which gives rise to fibrin deposits in lung alveoli of cattle with pasteurellosis (Cheryk *et al.*, 1998; Nyarko *et al.*, 1998;). As the concentration of leukotoxins increases, target cells are stimulated to undergo apoptosis, involving LFA-1 signalling through protein kinase C and adverse effects on the mitochondria (Kato *et al.*, 1995; Korostoff *et al.*, 2000; Kelk *et al.*, 2003; Atapattu & Czuprynski, 2005;). At higher leukotoxin concentrations, the apoptotic mechanisms are exceeded and necrosis occurs due to pore formation. LktA-mediated cell permeabilization was shown to cause a rapid leakage of the intracellular potassium and cell swelling (Clinkenbeard *et al.*, 1989a; Clinkenbeard & Upton, 1991;). *In vitro* studies showed that LtxA formed voltage-gated ion channels of large conductance in the planar lipid bilayer, with an approximate functional diameter of 0.96 nm (Iwase *et al.*, 1990; Lear *et al.*, 1995;). A pore size of about 1.2 nm was, in turn, deduced for LktA from osmotic protection experiments with raffinose (Clinkenbeard *et al.*, 1989b). In addition, metabolites from phospholipase C activation (arachidonic acid) appear to contribute to LktA-induced cytolysis significantly (Jeyaseelan *et al.*, 2001).

### AC toxin – a bifunctional toxin with a cell-invasive enzymatic and pore-forming activity

The 1706-residue-long CyaA is unique among RTX leukotoxins by being a bifunctional toxin in which a cell-invasive AC enzyme domain (∼400 residues) has been fused to the N-terminus of a pore-forming RTX ‘hemolysin’ moiety (Fig. 3). This part of CyaA (∼1300 carboxy-proximal residues) consists of typical segments, such as (1) a hydrophobic pore-forming domain comprising residues 500–800 (Benz *et al.*, 1994); (2) an activation domain, where the post-translational palmitoylation of lysine residues 860 and 983 of CyaA occurs (Hackett *et al.*, 1994, 1995); and (3) a typical calcium-binding RTX domain, harboring the nonapeptide repeats of a consensus sequence X-(L/I/F)-X-G-G-X-G-(N/D)-D, which form numerous (∼40) calcium-binding sites (Rose *et al.*, 1995; Rhodes *et al.*, 2001;).

CyaA primarily targets and paralyzes with high efficacy the leukocytes expressing the α_M_β_2_ integrin (CD11b/CD18). With reduced efficiency, however, the toxin can also penetrate and deliver the AC enzyme into a variety of cells lacking the CD11b/CD18 (Hanski, 1989; Bellalou *et al.*, 1990;). Unlike most other enzymatically active toxins, which penetrate into cell cytosol from endosomes, several reports showed that the AC is delivered directly across the cytoplasmic membrane of cells without the need for receptor-mediated endocytosis of the toxin (Gentile *et al.*, 1988; Gordon *et al.*, 1988; Guermonprez *et al.*, 1999; Schlecht *et al.*, 2004; Basler *et al.*, 2006;). In target cytosol, the N-terminal AC domain of CyaA binds intracellular calmodulin, whereupon its specific enzymatic activity is increased by ∼10 000-fold and catalyzes uncontrolled conversion of cellular ATP to cAMP, a key second messenger signalling molecule (Wolff *et al.*, 1980; Confer & Eaton, 1982;). This subverts the signalling of protein kinase A and essentially instantaneously ablates the bactericidal functions of phagocytes, such as oxidative burst and phagocytosis, and induces the secretion of immunomodulatory cytokines (Vojtova *et al.*, 2006). Recently, the crystal structure of the AC domain of CyaA in the complex with the C-terminal fragment of calmodulin was resolved by Guo *et al.* (2005).

The main segment of CyaA required for binding to the α_M_β_2_ integrin was located in the glycine-rich repeat region between residues 1166 and 1281 of CyaA (El-Azami-El-Idrissi *et al.*, 2003). However, cooperation and structural integrity of all domains of the ‘hemolysin’ moiety of CyaA appear to be critical for membrane insertion and translocation of the N-terminal AC enzyme domain into cell cytosol (Bellalou *et al.*, 1990; Iwaki *et al.*, 1995;). Translocation itself, but not the mere insertion of CyaA into the cytoplasmic membrane of cells, is driven by a negative membrane potential (Otero *et al.*, 1995). Fiser and colleagues showed that the translocating AC polypeptide inserted into the cell membrane participates in the formation of a novel type of membrane path for Ca^2+^ influx into monocytic cells. The latest results from our laboratory show that calcium influx induces talin cleavage by calpain and enables mobilization of CyaA with CD11b/CD18 into lipid rafts, where the cholesterol-enriched lipid environment supports the translocation of the AC domain across membrane (Bumba *et al.*, 2010). Moreover, translocation of the AC domain and oligomerization into cation-selective pores appear to represent two independent and parallel/competing activities of the membrane-inserted form of CyaA. Either activity can be upmodulated at the expense of the other by specific substitutions of key glutamate residues forming pairs in the predicted transmembrane segments between residues 500 and 700 of CyaA. These were found to play a critical role in cell binding, formation of cation-selective pores and translocation of the AC domain into the cells (Osickova *et al.*, 1999, 2010; Basler *et al.*, 2007; Fiser *et al.*, 2007; Vojtova-Vodolanova *et al.*, 2009). The ‘hemolysin’ pores formed by CyaA have a diameter of only about 0.6–0.8 nm and the specific ‘hemolytic’ activity of CyaA is relatively low, compared with HlyA for example (Bellalou *et al.*, 1990). It appears, nevertheless, to synergize with the invasive AC enzyme activity in maximizing the overall cytotoxic potency of the toxin on CD11b^+^ cells (Basler *et al.*, 2006; Hewlett *et al.*, 2006;).

The role of CyaA in the interaction of *Bordetella* with cells of the respiratory epithelia and in the modulation of the host immune response through the induction of proinflammatory cytokine secretion remains poorly explored. Recent work has suggested that CyaA activity may account for the induction of IL-6 in tracheal epithelia colonized by *B. pertussis* (Bassinet *et al.*, 2004). CyaA contributes to numerous pathological effects in the murine model of lung infection, such as efficient pulmonary colonization, induction of histopathological lesions in lungs, recruitment of inflammatory leukocytes and induction of lethality (Weiss *et al.*, 1984; Gueirard *et al.*, 1998;). Low concentrations (1–5 ng mL^−1^) of CyaA have recently been shown to effectively inhibit complement-mediated opsonophagocytosis, which is a crucial defense mechanism of naive unimmunized hosts (Kamanova *et al.*, 2008). In fact, CyaA-mediated intoxication by cAMP was also found to inhibit the phagocytosis of *B. pertussis* cells via Fc receptors by neutrophils (Weingart & Weiss, 2000). CyaA activity further causes a loss of chemotactic and oxidative burst capacities required for the bactericidal activity of leukocytes (Friedman *et al.*, 1987). Moreover, the toxin can induce macrophage apoptosis (Khelef *et al.*, 1993; Khelef & Guiso, 1995;) by a mechanism involving disruption of the membrane potential of mitochondria (Bachelet *et al.*, 2002). In many *Bordetella* isolates, the CyaA protein remains attached to the bacterial surface following secretion, due to interaction with the filamentous hemagglutinin (Zaretzky *et al.*, 2002). Most of the cell-attached CyaA appears, however, to be aggregated and unable to act as a ‘contact weapon’, because only the newly secreted CyaA was found to be capable of penetrating target cells and increasing the intracellular cAMP levels (Gray *et al.*, 2004).

#### Use of CyaA in research and vaccine applications

Because Gram-negative bacteria generally do not express calmodulin homologues and the AC domain exhibits only residual enzyme activity in the absence of calmodulin, the AC domain of CyaA could elegantly be used as a reporter enzyme for tracing protein translocation into the eukaryotic cell cytosol (Sory & Cornelis, 1994). The AC (Cya) reporter protein is fused to an effector protein secreted through the type III secretion pathway (TTSS), such as the *Yersinia* Yop proteins. While the fusion exhibits only very low AC enzyme activity in the bacterial cell and/or the culture supernatants, once it is injected through the TTSS pathway into a eukaryotic host cell, the AC enzyme is activated >1000-fold by host calmodulin and catalyzes the rapid conversion of ATP to cAMP. Use of the AC (Cya) fusion reporter has now become a standard technique for the demonstration of contact-dependent direct translocation of TTSS effector proteins into animal and plant host cells by a number of Gram-negative species.

Another original application makes use of the fact that the residual activity of the AC domain in the absence of calmodulin requires the physical interaction of the T25 and T18 fragments of the AC domain. This could be well exploited to develop a bacterial two-hybrid system for the detection of protein–protein interactions (Karimova *et al.*, 1998). When the T25 and T18 fragments are individually fused to peptides or proteins that are able to bind each other, the interaction of the chimeric polypeptides brings together the T25 and T18 fragments, resulting in the restoration of a residual capacity of the enzyme to convert intracellular ATP to cAMP, even in the absence of eukaryotic calmodulin. This, in turn, can be monitored in a rather sensitive manner using *E. coli* hosts lacking the endogenous AC activity as indicator strains for the two-hybrid screening, because even very low levels of cAMP produced by the reconstituted AC enzyme will allow transcription of the genes involved in lactose and maltose catabolism in *E. coli*.

In another application, the genetically detoxified CyaA, unable to increase cAMP levels, could be exploited for highly efficient *in vivo* delivery of foreign T-cell epitopes into the major histocompatibility complex class I- and II-dependent antigen presentation pathways of CD11b^+^ dendritic cells (Osicka *et al.*, 2000; Loucka *et al.*, 2002; Schlecht *et al.*, 2004;). This allowed the use of CyaA for antigen delivery and induction of strong Th1-polarized and epitope-specific CD8^+^ cytotoxic T-cell responses, effective in prophylactic vaccination against viruses and in immunotherapy of certain tumors (Sebo *et al.*, 1995; Fayolle *et al.*, 1996, 1999, 2001; Saron *et al.*, 1997; Dadaglio *et al.*, 2000; Loucka *et al.*, 2002; Preville *et al.*, 2005; Mackova *et al.*, 2006). Phase I/II human clinical trials aimed at exploring this exciting application of CyaA for cervical cancer and melanoma immunotherapy are currently in preparation.

### MARTX

A quite different and recently discovered division of the RTX-toxin family is a group of very large toxins that differ from all previously known RTX proteins by the molecular structure and *rtx* gene cluster organization (Table 1, Fig. 3). These MARTX have thus far been identified in several different *Vibrio* species (*Vibrio* sp. RC385, *V. cholerae, Vibrio splendidus, Vibrio anguillarum, Vibrio vulnificus*) and are also present in *Aeromonas hydrophila, Yersinia enterocolitica* and *Yersinia kristensenii, Proteus mirabilis, Photorhabdus luminescens* and *Photorhabdus asymbiotica* (Supporting Information, Tables S1 and S2) (Satchell, 2007; Li *et al.*, 2008;). The best-studied prototype of MARTX is the *V. cholerae* RtxA (VcRtxA), renamed to MARTX_*Vc*_ (Satchell, 2007).

All *rtxA*-like MARTX genes encode proteins that range from 3212 to 5206 amino acid residues in length. In contrast to other toxins, the C-terminal repeats of MARTX proteins exhibit an 18-residue-long consensus sequence X(V/I)XXGXXNX(V/I)XXGDGXDX. These share a common G-7X-GXXN central motif, instead of the typical nonapeptide repeat (Lin *et al.*, 1999; Satchell, 2007;). Moreover, MARTX proteins possess additional N-terminal repeats, which fall in two classes. The first has a 20-residue consensus sequence GXXG(N/D)(L/I)(T/S)FXGAG(A/G)XNX(L/I)X(RH) and the second has a 19-residue consensus T(K/H)VGDGX(S/T)VAVMXGXAN(I/V)X. Altogether, the glycine-rich repeats represent about 25% of the sequence of MARTX*_Vc_* and were proposed to bind the eukaryotic cell surface and to facilitate the translocation of a central ∼1700 amino acid portion of the MARTX*_Vc_* to the target cell cytosol. These central regions of MARTX are composed of differing activity domains that, upon entry into the eukaryotic cell, may exert different cytotoxic activities (Boardman & Satchell, 2004; Satchell, 2007;). However, except for the central domains of MARTX*_Vc_* and part of *V. vulnificus* RtxA (MARTX*_Vv_*), the biological activity and function of these large RTX proteins remain unknown. Little is known about the post-translational modifications of MARTX proteins. An *rtxC* gene similar to the *hlyC* acyltransferase gene required for maturation of *E. coli*α-hemolysin is located in the same operon with the *rtxA* gene (Lin *et al.*, 1999). The recent data indicate that *rtxC* is not necessary for the MARTX toxin function in *V. cholerae*, but the MARTX toxin without *rtxC* activation showed reduced actin cross-linking activity (Cheong *et al.*, 2010), while a deletion of *rtxC* had no effect on the virulence of *V. vulnificus* (Lee *et al.*, 2007; Liu *et al.*, 2007;). These results suggest that acylation may not be essential for all MARTX toxins (Satchell, 2007).

Analysis of the available genomic structures revealed that the *martx* loci typically consist of two divergent operons (*rtxHCA* and *rtxBDE*). The toxin gene is found as the third gene downstream of the *rtxC* homologue (a putative acyl transferase) and a conserved hypothetical gene (*rtxH*) of unknown function, which is found only in the *martx* gene clusters. The divergent operon contains three more genes that encode homologues of the ATP-binding transporter protein RtxB, the MFP RtxD and a second ATPase, RtxE (Boardman & Satchell, 2004). The latter also appears to be involved in MARTX secretion by TISS, as *rtxE* gene disruption in *V. vulnificus* and *V. anguillarum* blocked the secretion of MARTX*_Vv_* (MARTX*_Va_*, respectively) and induced a significant reduction in bacterial cytotoxic activity against epithelial cells *in vitro* (Lee *et al.*, 2008; Li *et al.*, 2008;). The necessary homologues of the OMP *tolC* are then found outside the *rtx* loci. This atypical four-component TISS seems to be a conserved feature across the entire MARTX family (Boardman & Satchell, 2004).

The production of MARTX TISS components is regulated by the growth phase. The repressor regulating *rtxBDE* expression is encoded outside the *rtx* locus and is not directly linked to quorum sensing, while *V. cholerae* may apparently couple the regulation of the *rtx* locus to the detection of stress (Boardman *et al.*, 2007). The *rtx*H, *rtx*C and *rtx*A genes are coordinately expressed on a single mRNA (Boardman *et al.*, 2007).

The prototype *rtxA* gene was found in both clinical and environmental isolates of *V. cholerae*, but not in the O1 classical biotypes (Lin *et al.*, 1999; Chow *et al.*, 2001;). The deduced MARTX*_Vc_* protein is 4545 residues long, with a predicted molecular mass of >485 kDa (Lin *et al.*, 1999). In contrast to pore-forming RTX leukotoxins, the MARTX*_Vc_* does not appear to disrupt membrane integrity or cause cell death. Rather, MARTX*_Vc_* activity contributes to the severity of acute inflammatory responses in the pathology of cholera by inducing alteration of permeability of the paracellular tight junctions. This results from the capacity of MARTX*_Vc_* to induce cell rounding and depolymerization of the actin cytoskeleton in a broad range of cell types, and yet the cells remain viable (Cordero *et al.*, 2006). Concurrent with actin stress fiber disassembly, actin monomers are covalently cross-linked into dimers, trimers and higher multimers by the actin cross-linking domain (ACD), which utilizes G-actin as a substrate and hydrolyzes one molecule of ATP per cross-linking event (Kudryashov *et al.*, 2008a). The ACD of MARTX*_Vc_*, located between residues 1963 and 2375, catalyzes a unique reaction consisting of the formation of an intermolecular iso-peptide bond between the γ-carboxyl group of glutamic acid residue 270 and the ɛ-amino group of lysine residue 50 of actin (Kudryashov *et al.*, 2008b). Contrary to expectation, however, deletion of the ACD did not ablate the cell-rounding activity of MARTX*_Vc_*, revealing that the large toxin carried a second cell-rounding activity (Lin *et al.*, 1999; Fullner & Mekalanos, 2000; Fullner *et al.*, 2001, 2002; Sheahan *et al.*, 2004). This targets the regulation of the small Rho GTPases, Rho, Rac and Cdc42, rather than the Rho GTPase proteins directly, and a 548-residue-long Rho inactivation domain (RID) of MARTX*_Vc_* was recently found to inactivate the Rho GTPases by a mechanism distinct from other Rho-modifying bacterial toxins (Sheahan & Satchell, 2007).

The MARTX*_Vc_* toxin was found to insert into the host cell plasma membrane and is supposed to directly translocate the ACD to the cytosol of cells in a way involving insertion of the N- and C-terminal repeat regions into the cell membrane. Inside the cell, the ACD is released into cell cytosol (Sheahan *et al.*, 2004) through self-processing catalyzed by a conserved cysteine protease domain (CPD), which cleaves MARTX*_Vc_* between residues L^3428^ and A^3429^. Three additional CPD-dependent processing sites were identified, all at leucine residues delimiting the ACD, RID and α/β junction domains (Prochazkova *et al.*, 2009; Shen *et al.*, 2009;). Autoprocessing of MARTX*_Vc_* thereupon results in the release of individual activity domains into target cytosol. Autoprocessing activity of the CPD is induced by binding inositol hexakisphosphate (InsP_6_), which is abundant at the inner surface of the cell membrane (Prochazkova & Satchell, 2008), and the CPD activation mechanism was recently characterized in molecular detail (Prochazkova *et al.*, 2009). Because InsP_6_ is exclusive to eukaryotes and is present at cytosolic concentrations >10 μM, the evolution of a proteolytic biosensor responding to InsP_6_ appears to be an ingenious strategy for assuring that the functional activation of a secreted toxin occurs only once it has reached the host cell cytosol (Lupardus *et al.*, 2008).

The sequence of the *V. vulnificus* RTX toxin (VvRtxA, MARTX_*Vv*_) was reported in 2003 (Chen *et al.*, 2003), showing that it consists of 5206 residues and has a predicted molecular mass of 556 kDa (Lee *et al.*, 2007). The deduced primary amino acid sequence of MARTX_*Vv*_ is ∼80–90% identical throughout most regions to that of MARTX*_Vc_*. However, no ACD is present in MARTX*_Vv_*, which also does not cause actin cross-linking (Sheahan *et al.*, 2004) and posesses only the Rho-inactivating activity associated with an RID (Sheahan & Satchell, 2007). This is followed by a CPD located towards the C-end of MARTX*_Vv_* (Prochazkova & Satchell, 2008).

Unlike MARTX*_Vc_*, the MARTX*_Vv_* may be able to disrupt membranes by the predicted segments homologous to those of pore-forming RTX toxins. This would go well with the difference in the virulence of the pathogens, where *V. vulnificus* is exceedingly more destructive and cytolytic, as compared to *V. cholerae* (Lee *et al.*, 2007). Indeed, MARTX*_Vv_* appears to be crucial for *V. vulnificus* virulence and cytotoxicity (Kim *et al.*, 2007; Lee *et al.*, 2007; Liu *et al.*, 2007;).

The RtxA of *V. anguillarum* appears to play a major role in the virulence of the agent causing vibriosis in fish. The *rtx* operon encodes a second hemolysin gene cluster in *V. anguillarum* M93Sm, which also has a hemolysin gene, *vah1*. While Vah1 causes cell vacuolation, the MARTX*_Va_* causes cell rounding. Analysis of the MARTX*_Va_* sequence reveals that the protein does not contain an ACD, but a homologue of the RID was identified. The contribution of the RID to cell rounding needs to be investigated further. Single mutations in *vah1* or *rtxA* attenuate the cytotoxicity of *V*. *anguillarum* M93Sm and a *vah1+rtxA* double mutant is no longer cytotoxic (Li *et al.*, 2008).

The genes for MARTX homologues of *P. luminescens* are clustered in two chromosomal regions and are tandemly organized. There are four loci containing intact *rtxA* genes, while four are disrupted by frameshifts or insertion sequences. The organization of genes encoding the RTX secretion system is identical to that of *V. cholerae* (Duchaud *et al.*, 2003). The function of these genes in pathogenesis was not analyzed, but the actin cross-linking domain was not found in MARTX sequences from *Photorhabdus*, which appear to have evolved unique cellular activities through the acquisition of a different genetic material (Sheahan *et al.*, 2004).

### Cadherin domain proteins

A particularly novel class of RTX proteins of *Vibrio* is represented by RtxL1 and RtxL2, which are characterized by the presence of more than one cadherin domain and were first identified in *V. cholerae* N16961 (Chatterjee *et al.*, 2008). The *rtxL1* and *rtxL2* genes are arranged in tandem, which is different from the arrangement of the *V. cholerae* RTX gene cluster (Lin *et al.*, 1999). RtxL1 and RtxL2 belong to the RTX family of hemolysin/leukotoxins, exhibiting hemolytic activity on human erythrocytes, but also appear to play a role in adherence and biofilm formation by *V. cholerae* N16961.

Both *rtxL1* and *rtxL2* genes are expressed in all *V. cholerae* isolates belonging to O1 (strains N16961, O395 and 569B), O139 (strain SG24) and non-O1-nonO139 (strains VCE232 and VCE309) serovars under *in vitro* conditions and appear to play a role in virulence in a mouse model (Chatterjee *et al.*, 2008).

Another member of the RTX-toxin group with cadherin domains was found as a novel RTX-like hemagglutinin (FrhA) of the *V. cholerae* O1 classical strain O395, the O1 El Tor strain A1552 and the O1 El Tor strain P27459 (Syed *et al.*, 2009). FrhA expression is positively regulated by the flagellar regulatory hierarchy. It mediates adherence to chitin and epithelial cells, enhances biofilm formation and is involved in intestinal colonization in infant mice.

### RTX proteases

The RTX proteases belong to microbial zinc metalloproteases (Hooper, 1994; Miyoshi & Shinoda, 2000;) and form a group of approximately 50 kDa proteolytic enzymes secreted by a variety of pathogens. These proteases consist of an N-terminal proteolytic domain and a C-terminal calcium-binding RTX domain and are synthesized as zymogens that are activated by processing upon secretion.

The RTX proteases belong to the subgroup of metzincin metalloendopeptidases (Stocker & Bode, 1995; Stocker *et al.*, 1995;) that contains an extended zinc-binding motif HEXXHXXGXXH, of which the three histidine residues are involved in binding the catalytically essential zinc ion, while the glutamic acid residue was postulated to take part in the catalytic activity. In addition, the metzincins all share a conserved methionine, which is located on a turn near the catalytic site, some 40–60 residues towards the C-terminus (the Met-turn).

To date, these proteases with characteristics of the RTX protein family were discovered and identified in six bacterial genera (Table 2): *Serratia* (Nakahama *et al.*, 1986), *Erwinia* (Wandersman *et al.*, 1987; Dahler *et al.*, 1990; Letoffe *et al.*, 1990; Zhang *et al.*, 1999;), *Pseudomonas* (Guzzo *et al.*, 1990; Duong *et al.*, 1992; Liao & McCallus, 1998; Chabeaud *et al.*, 2001; Woods *et al.*, 2001;), *Proteus* (Wassif *et al.*, 1995; Walker *et al.*, 1999;), *Caulobacter* (Umelo-Njaka *et al.*, 2002) and *Photorhabdus* (Bowen *et al.*, 2003).

**Table 2:** Known members of the RTX protease family

Bacterium	Described RTX proteases
*Serratia marcescens*	PrtSM
*Erwinia chrysanthemi*	PrtG, PrtB, PrtC, PrtA
*Erwinia amylovora*	PrtA
*Pseudomonas aeruginosa*	AprA
*Pseudomonas fluorescens*	AprX
*Pseudomonas brassicacearum*	AprA
*Proteus mirabilis*	ZapA, ZapE
*Caulobacter crescentus*	Sap
*Photorhabdus*	PrtA

### Genetic organization of RTX protease loci

The RTX protease locus of *P. aeruginosa* alkaline protease AprA was first identified by Duong in 1992 (Duong *et al.*, 1992). It consists of five ORFs, with *aprD, aprE* and *aprF* encoding the TISS proteins, the structural *aprA* gene for the protease and *aprI* encoding a protease inhibitor (inh).

**Fig. 4:**
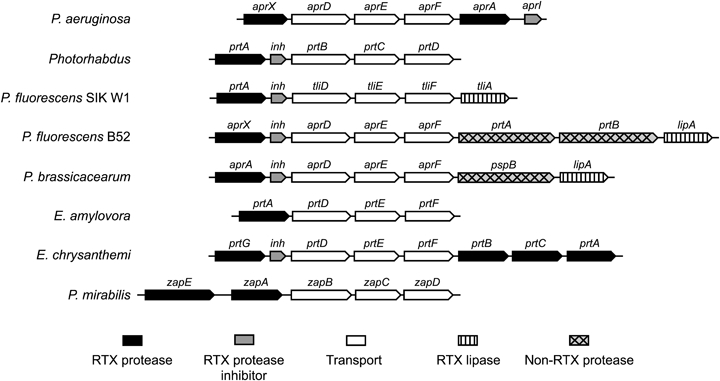
The schematic representation of *rtx* protease gene clusters. The arrows represent coding regions and transcriptional directions of the genes deposited under the GenBank accession numbers: *Pseudomonas aeruginosa* (AY003006, X64558), *Photorhabdus* (AY230750), *Pseudomonas fluorescens* SIK W1 (AF083061), *P. fluorescens* B52 (AF216700, AF216701, AF216702), *Pseudomonas brassicacearum* (AF286062), *Erwinia amylovora* (Y19002), *Erwinia chrysanthemi* (M60395), *Proteus mirabilis* (AF064762).

The genetic organization of RTX protease operons varies. In *Pseudomonas fluorescens* CY091 (Liao & McCallus, 1998), the protease gene is located upstream of the *inh* and *aprDEF* transporter genes. The same organization of the locus was shown in *P. luminescens* (Bowen *et al.*, 2003), starting with the structural *prtA* gene for the protease, and followed by a gene encoding a putative protease inhibitor, *inh* and then the three members of an associated TISS, *prtB, prtC* and *prtD* (Fig. 4).

The genetic organization of RTX protease operons appears to depend on the number of secreted proteases. For example, *E. chrysanthemi*, a phytopathogenic enterobacterium, secretes four RTX proteases, where the first gene in the operon is the structural gene for *prtG* (Ghigo & Wandersman, 1992). This is followed by a gene encoding a putative protease inhibitor, *inh*, and then the three genes encoding TISS components, *prtD, prtE* and *prtF*. Structural genes for the other three RTX proteases *prtB, prtC* and *prtA* are adjacent to and belong to independent transcription units (Delepelaire & Wandersman, 1989; Dahler *et al.*, 1990; Ghigo & Wandersman, 1992;).

Unlike *E. chrysanthemi*, the other phytopathogenic species, *Erwinia amylovora*, secretes a single protease (Zhang *et al.*, 1999), while *P. mirabilis* produces two RTX proteases: ZapA and ZapE (Walker *et al.*, 1999). ZapE shares homology with ZapA and other RTX proteases, but is larger (687 residues).

In some bacterial species, the RTX protease operon is combined with the genes for RTX lipases or other proteins. For example, in *P. fluorescens* B52, an *aprX*–*lipA* operon contains the protease (*aprX*) and the lipase (*lipA*) genes encoded at opposite ends of a contiguous set of genes (Woods *et al.*, 2001). This also includes the protease inhibitor, TISS genes and two autotransporter genes (*aprX*-*inh*-*aprDEF*-*prtAB*-*lipA*), constituting an operon (Ahn *et al.*, 1999).

### Structure–function relationships of RTX proteases

The typical size of RTX proteases is around 480 amino acid residues and 50 kDa, with the theoretical isoelectric point of most RTX proteases being about 4.0–4.6. Delepelaire & Wandersman (1989) first showed that the extracellular proteases B and C of *E. chrysanthemi* were synthesized as inactive zymogens proB and proC that were activated by autoprocessing of 16 or 18 amino acid residues (∼2 kDa) from the N-termini in the external medium containing divalent cations. As other zinc metalloproteases, the RTX proteases are inhibited by the general metalloprotease inhibitor EDTA, as well as by *o*-phenanthroline, a specific zinc metalloprotease inhibitor (Zhang *et al.*, 1999).

Three-dimensional structures of the alkaline proteases of *P. aeruginosa* (AprA) and PrtSM of *S. marcescens* were the first RTX protein structures resolved (Baumann *et al.*, 1993; Baumann, 1994;). Both proteases have a very similar two-domain structure. The N-terminal part is the proteolytic domain with a folding topology very similar to astacin, the archetypical metzincin protease (Bode *et al.*, 1996). The C-terminal RTX domain consists of a 21-strand β sandwich. Within this domain, a ‘parallel β roll’ structure was first described, in which successive β strands are wound in a right-handed spiral and Ca^2+^ ions are bound within the turns between strands by a repeated GGXGXD sequence, thus setting a paradigm for the RTX portions of the whole protein family.

### RTX protease inhibitors

The RTX protease inhibitor from *E. chrysanthemi* (Inh) was shown to be synthesized as a 12 kDa polypeptide having a signal peptide of 19 residues, which is cleaved off during Sec-dependent secretion into the periplasm (Letoffe *et al.*, 1989). The mature 10 kDa inhibitor is entirely located in the periplasm of *E. chrysanthemi* and its presumed physiological role consists in protecting periplasmic proteins against proteases that might potentially leak out from the TISS channel assembly. Inh forms a 1 : 1 complex with proteases A, B and C from *E. chrysanthemi*, AprA from *P. aeruginosa* and PrtSM from *S. marcescens* (Letoffe *et al.*, 1989). In the crystal structure of the complex of *S. marcescens* PrtSM with Inh from *E. chrysanthemi* (Baumann *et al.*, 1995), the Inh was found to fold into a compact eight-stranded antiparallel β-barrel structure, interacting with the protease via five N-terminal residues that insert into the active site cleft of PrtSM.

Similar inhibitors have been characterized for other RTX proteases, such as *P. aeruginosa* (Feltzer *et al.*, 2000), *S. marcescens* (Kim *et al.*, 1995) and *Photorhabdus* (Valens *et al.*, 2002; Bowen *et al.*, 2003;). The SmaPI inhibitor of *S. marcescens*, however, shows a very high protease specificity, while the protease inhibitor of *P. aeruginosa* (APRin) exhibits a significantly higher inhibitory activity (*K*_D_ of 4 pM) compared with the inhibitors of *E. chrysanthemi* and *S. marcescens* (*K*_D_ values from 1 to 10 μM).

### Biological activity of RTX proteases

*Pseudomonas aeruginosa* AprA was shown to hydrolyze fibrin and fibrinogen, with specific activities similar to plasmin (Shibuya *et al.*, 1991). AprA also exhibits an anticoagulant activity in human plasma, which was attributed to its direct fibrinogenolytic function. This may account, at least in part, for the most characteristic pathologic feature of the *P. aeruginosa* septicemia, which consists in hemorrhagic lesions lacking thrombi (Fetzer *et al.*, 1967). Purified *P. aeruginosa* AprA also readily cleaves soluble laminin (Heck *et al.*, 1986), which suggests its direct role (together with elastase) in both tissue invasion and hemorrhagic tissue necrosis in *P. aeruginosa* infections. Further, AprA also degrades human γ-interferon and inhibits its biological activity (Horvat & Parmely, 1988). Moreover, the effects of *P. aeruginosa* AprA on serum complement and on the isolated components C1q and C3 were also described (Hong & Ghebrehiwet, 1992). Because both C1q and physiological fragments of C3 (C3b, iC3b, and C3dg) are important opsonins, degradation of these molecules by *Pseudomonas* enzymes may facilitate the survival and proliferation of the organism in plasma.

The RTX protease from *P. aeruginosa* (Kreger & Gray, 1978) also appears to participate in liquefactive necrosis of the cornea through the digestion of the proteoglycan extracellular matrix, a major structural component of the cornea.

The ZapA protease, secreted by the urinary tract pathogen *P. mirabilis*, was found to cleave immunoglobulin G (IgG) (Loomes *et al.*, 1993) and both IgA1 and IgA2 (Wassif *et al.*, 1995), potentially conferring protection against opsonization of the bacterium during urinary tract infections.

The role of PrtA in the virulence of the bacterial entomopathogen *Photorhabdus* awaits clarification. The bacterium lives in symbiosis with nematodes that invade insects. Following entry into the insect, the bacteria are released from the nematode gut into the open blood system of the insect. Here, they secrete factors that kill the host and digest host tissues into nutrients for the replicating bacteria and nematodes. The secreted RTX protease PrtA of *Photorhabdus*, however, was not shown to be any major virulence factor (Bowen *et al.*, 2003) and may play an alternative role in the host bioconversion.

*Caulobacter crescentus* synthesizes another unusual type of RTX protease (Umelo-Njaka *et al.*, 2002) called Sap (S-layer-associated protease). The N-terminal half of Sap exhibits significant similarity to other RTX proteases (e.g. AprA of *P. aeruginosa*), including the characteristic RTX repeat sequences, while the C-terminal half of Sap exhibits a significant similarity to the N-terminal region of the S-layer protein RsaA. The hypothesis is that Sap evolved by combining the catalytic portion of an RTX protease with an S-layer-like protein, perhaps to associate with nascent S-layer monomers to scan for modifications. Despite no clearly identifiable type I secretion signal, Sap still appears to be secreted by a TISS (Ford *et al.*, 2007).

### RTX bacterial lipases

Extracellular lipases of Gram-negative bacteria have been extensively characterized, being considered as valuable tools for a variety of biotechnological, biomedical and food industry applications (Jaeger *et al.*, 1994). Until now, the genera *Pseudomonas* and *Serratia* were reported to produce RTX lipases of the I.3 subfamily. These do not have cysteine residues, do not require any additional gene products for activity and are secreted through TISS (Duong *et al.*, 1994; Akatsuka *et al.*, 1995; Ahn *et al.*, 1999;). Studies were conducted on lipases from *P. fluorescens* strain B52, SIK W1, no. 33, LS107d2, HU380 (Chung *et al.*, 1991; Johnson *et al.*, 1992; Tan & Miller, 1992; Kumura *et al.*, 1998; Kojima & Shimizu, 2003; Kojima *et al.*, 2003; Jiang *et al.*, 2005;), *S. marcescens* strains SM6, Sr41 (Akatsuka *et al.*, 1994; Li *et al.*, 1995;), *Pseudomonas brassicacearum* (Chabeaud *et al.*, 2001) and *Pseudomonas* sp. strains MIS38 and KB700A (Amada *et al.*, 2000; Rashid *et al.*, 2001;). The polyester polyurethanases PueA and PueB of *Pseudomonas chlororaphis* are also classified as lipases (Stern & Howard, 2000).

Lipases from *P. fluorescens* show remarkable sequence similarity to that of *S. marcescens*, with an identity of about 65% over virtually the entire length of the sequence (Li *et al.*, 1995). The *S. marcescens* SM6 and *Pseudomonas* sp. MIS38 enzymes are 613 and 617 residues long, respectively, and compared with *P. fluorescens* lipase, which is 476 amino acids in length, appear to bear an extra domain consisting of 138 and 135 amino acid residues inserted between residues Asn^405^ and Thr^406^ of the *P. fluorescens* lipase backbone (Amada *et al.*, 2000).

Lipase production by *P. fluorescens* B52 was shown to be repressed by iron and is regulated by temperature. Optimal lipase production occurs well below the optimal growth temperature (Woods *et al.*, 2001). Lipase production is also regulated by the homologue of the *E. coli* EnvZ-OmpR two-component osmoregulatory system and its secretion is reduced by NaCl (McCarthy *et al.*, 2004). The lipase production by *P. brassicacearum* was, in turn, shown to be under the control of phase variation (McCarthy *et al.*, 2004).

**Fig. 5:**
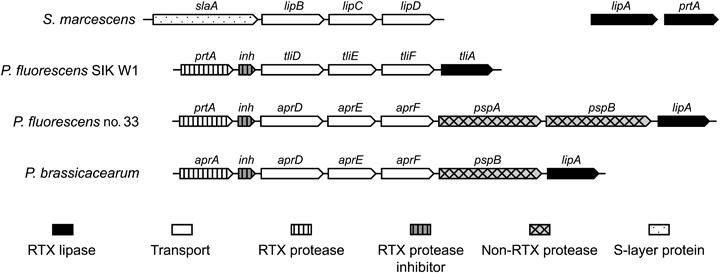
The schematic representation of *rtx* lipase gene clusters of lipases. The arrows represent coding regions and transcriptional directions of the genes deposited under the GenBank accession numbers: *Serratia marcescens* (D49826), *Pseudomonas fluorescens* SIK W1 (AF083061), *P. fluorescens* no. 33 (AB015053), *Pseudomonas brassicacearum* (AF286062).

In *S. marcescens*, the *lipA* gene is not linked to TISS component genes for LipB (ABC protein), LipC (MFP) and LipD (OMP), which, besides secreting LipA, can also promote the secretion of the metalloprotease, PrtA (Nakahama *et al.*, 1986), and of the S-layer protein homologue SlaA (Kawai *et al.*, 1998). In *P. fluorescens* SIK W1, the RTX lipase gene *tliA* is situated downstream of the ABC exporter genes *tliDEF*, with genes *prtA* and *inh*, for an RTX protease and a protease inhibitor, being located upstream of *tliDEF* (Ahn *et al.*, 1999). In *P. fluorescens* strain no. 33, *lipA* is clustered with an *aprA* gene for an alkaline protease, *aprDEF* genes for the TISS and *pspA* and *pspB* genes for two homologues of *Serratia* serine proteases (Kawai *et al.*, 1999). In the *P. fluorescens* B52, *lipA* is situated downstream of the *aprX*-*inh*-*aprDEF*-*prtAB* operon (Woods *et al.*, 2001). The organizations of the various gene clusters are depicted in Fig. 5.

### RTX bacteriocins

Bacteriocins are structurally and functionally diverse bacterial toxins inhibiting the growth of other bacterial strains (Jacob *et al.*, 1973). Plasmid-borne genes for bacteriocins belonging to the RTX protein family (Table 3) were found in Gram-negative plant endosymbionts and pathogens, such as *Rhizobium leguminosarum, Bradyrhizobium elkanii, Xylella fastidiosa, Xanthomonas oryzae* or *Agrobacterium tumefaciens*, respectively (Oresnik *et al.*, 1999; Simpson *et al.*, 2000; Venter *et al.*, 2001; Watson *et al.*, 2001; Cherif *et al.*, 2006; Sugawara *et al.*, 2007;). The mechanism of action of these putative bacterial toxins remains to be characterized. Nodulation competition experiments with mutants lacking the RTX bacteriocin activity indicated a role in the competitiveness of *R. leguminosarum* 248 (Oresnik *et al.*, 1999).

**Table 3:** Known members of the RTX bacteriocin family

Protein	Bacterium	Molecular weight (kDa)	Number of RTX repeats
ORF	*Rhizobium leguminosarum* strain 248	102	18
RzcA	*Rhizobium leguminosarum* bv. *viciae* strain 306	439	18
RtxA	*Bradyrhizobium elkanii*	88	NA
XF2407	*Xylella fastidiosa*	219	NA
XF2759	*Xylella fastidiosa*	139	NA
RtxA	*Xanthomonas oryzae* pv. *oryzae*	48	NA
RzcA	*Agrobacterium tumefaciens* strain C58	204	54

### S-layer RTX proteins

S-layer proteins form regularly arranged two-dimensional crystalline arrays covering the entire outer surface of a broad spectrum of bacteria and archaea. These are often composed of a single protein or glycoprotein species of 40–200 kDa, which is endowed with the ability to assemble on the supporting envelope layer, thus representing one of the simplest self-assembly systems (Sleytr & Beveridge, 1999; Sleytr *et al.*, 2007;).

Several RTX proteins have been identified among S-layer proteins of pathogenic as well as nonpathogenic bacteria and cyanobacteria (see the section on RTX proteins involved in the motility of *Cyanobacteria* for more details). S-layer proteins have an acidic pH, lack cysteines and are produced in large quantities (10–12% of cell protein). They usually possess two structurally different domains, with the N-terminal part typically showing similarity to other S-layer proteins and the C-terminal part containing the characteristic RTX structures and an uncleaved TISS signal, respectively. The RTX repeats of S-layer proteins were shown to bind calcium, which was proposed to mediate proper S-layer crystallization.

The best-studied S-layer RTX protein is the 98 kDa RsaA that forms a hexagonal lattice on the cell surface of *C. crescentus*. Six copies of RsaA form a ring-like subunit that interconnects with other subunits to form a two-dimensional array consisting of approximately 40 000 RsaA units and with a porosity predicted to exclude molecules larger than ∼17 kDa (Smit *et al.*, 1992). Some RsaA monomers are anchored to the outer bacterial membrane via interaction with smooth lipopolysaccharide, while the others are presumed to remain surface-associated by interaction with already attached RsaA monomers (Bingle *et al.*, 1997a; Awram & Smit, 2001; Ford *et al.*, 2007;). Calcium appears to mediate proper RsaA crystallization and perturbations in RsaA at or near the RTX repeats result in the shedding of RsaA (Walker *et al.*, 1994; Beveridge *et al.*, 1997; Nomellini *et al.*, 1997; Ford *et al.*, 2007;). It is presumed that a key function of the S-layer is to form a selective porosity barrier protecting the bacterium from a variety of predatorial assaults in the complex environments of bacterial biofilms. The S-layer appears to provide a barrier protecting against attack by the predatory *Bdellovibrio*-like bacterium (Koval & Hynes, 1991).

The N-terminal part of the 98 kDa RsaA protein shows the highest homology score to *Campylobacter fetus* S-layer protein, while its C-terminal portion contains five RTX repeats (Gilchrist *et al.*, 1992). RsaA is produced constitutively to make up 10–12% of the total cell proteins, whereby it largely exceeds the secretion levels of any other type of RTX proteins. The ABC and MFP components of the TISS complex are encoded by *rsaD* and *rsaE* genes, located immediately downstream of the *rsaA* gene (Awram & Smit, 1998), while the *rsaF_a_* gene encodes one of two alternative OMP components of the RsaA secretion machinery and is located several kilobases downstream of the *rsaA* gene. The *rsaF_b_* gene encoding a second OMP component is completely unlinked. This is the only known example of a TISS that can utilize either of the two OMPs for the secretion of the same protein. Both OMPs, however, appear to be needed to handle the large amounts of RsaA produced, as neither one of the OMPs alone could induce wild-type secretion levels of RsaA (Toporowski *et al.*, 2004).

Because of high secretion levels, cell surface location and geometrical packing of the RsaA protein, and thanks to the ease of genetic manipulation, the *Caulobacter* S-layer system has been exploited for biotechnology applications. These comprise the secretion of large quantities of ‘passenger’ proteins of economic and research interest into culture media (Bingle *et al.*, 2000), or surface presentation of heterologous protein inserts on the the S-layer, such as S-layer-mediated display of the IgG-binding domain of streptococcal protein G (Bingle *et al.*, 1997b; Umelo-Njaka *et al.*, 2001; Nomellini *et al.*, 2007;).

Three other S-layer RTX proteins, Crs (1361 residues), CsxA (1123 residues) and CsxB (1238 residues), have also been identified in *Campylobacter rectus*, a Gram-negative bacterium associated with several forms of human periodontal disease (Miyamoto *et al.*, 1998; Wang *et al.*, 1998; Braun *et al.*, 1999; LaGier & Threadgill, 2008;). These proteins appear to be the potential virulence factors involved in the evasion of host defense, such as phagocytic uptake and bactericidal activity of serum (Okuda *et al.*, 1997; Thompson, 2002;).

SlaA, another S-layer family RTX protein of 1002 residues partially similar to the *C. crescentus* RsaA protein, was found in *S. marcescens* (Kawai *et al.*, 1998). The 101 kDa protein appears to be exported by the Lip TISS system and a sequence dissimilarity in the N-terminal regions of SlaA has been observed among different strains of *S. marcescens*, which may be related to the antigenic variation of *S. marcescens* (Kawai *et al.*, 1998).

### RTX proteins involved in the motility of *Cyanobacteria*

RTX proteins appear to be abundant in cyanobacteria, while the function of most of them remains elusive. Quite unexpectedly, however, some of the described cyanobacterial RTX proteins have been shown to be involved in cell motility, including SwmA of *Synechococcus* sp. strain WH8102, oscillin of *Phormidium uncinatum* or hemolysin-like protein Sll1951 of *Synechocystis* sp. strain PCC 6803.

The RTX protein SwmA was suggested to be part of the S-layer and to be required for the swimming motility of the marine unicellular cyanobacterium *Synechococcus* sp. strain WH8102 (Brahamsha, 1996; McCarren *et al.*, 2005;). Swimming *Synechococcus* strains are observed to rotate around their longitudinal axis, as they translocate at speeds of up to 25 μm s^−1^ and once fortuitously attached to a microscope slide, they rotate around the point of attachment. Intriguingly, cells with an insertionally inactivated *swmA* gene lack the S-layer and are nonmotile, and yet still rotate around the point of attachment. Thus, SwmA is somehow required for the generation of thrust, but not torque. However, the mechanism by which the SwmA and the S-layer function in motility remains elusive (Brahamsha, 1996; McCarren *et al.*, 2005;).

SwmA is a 130 kDa polypeptide that appears to be glycosylated and contains glycine- and aspartate-rich repeats. It is assumed that RTX repeats of SwmA function in calcium ion bridging to the outer membrane and thereby mediate the anchoring of the S-layer. Treatment of *Synechoccocus* sp. strain WH8102 with the chelator EDTA, indeed, removes the outer membrane and solubilizes SwmA (Brahamsha, 1996; McCarren *et al.*, 2005;).

The surface fibrils on the top of *P. uncinatum* S-layer consist of a single rod-shaped RTX protein of 646 residues with 46 repeats, the oscillin. Its structure appears to favor gliding, a relatively slow and smooth surface-associated translocation (Hoiczyk & Baumeister, 1997). As proposed by Hoiczyk and colleagues, the highly glycosylated surface of oscillin fibrils possesses ideal physicochemical properties for the temporary adhesion of the slime necessary for the generation of thrust and the helical arrangement of oscillin fibrils might guide the rotation of the *P. uncinatum* filament (Hoiczyk & Baumeister, 1997; Hoiczyk, 2000;).

The hemolysin-like RTX protein Sll1951 of 1741 residues produced by a unicellular freshwater cyanobacterium *Synechocystis* appears to be related to the elimination of motility, although the mechanism of its action remains largely unknown (Sakiyama *et al.*, 2006).

### Nodulation RTX proteins

A 30 kDa RTX protein, NodO, was identified in *R. leguminosarum* bv. *viciae* and was shown to play a role in pea and vetch nodulation (Economou *et al.*, 1990). The exact mechanism of the action of NodO in the process of the formation of nitrogen-fixing nodules on legume roots remains to be clarified. NodO was shown to form cation-selective channels in planar lipid bilayers (Sutton *et al.*, 1994), and two hypotheses were proposed on how this might enhance nodulation. One assumes that NodO pores in root cell membrane facilitate the passage of lipooligosaccharide nodulation factors. Another possibility could be the synergy of signalling resulting from cation fluxes through NodO channels across the plasma membrane, such as Ca^2+^ entry into root cells, thus amplifying the response induced by lipooligosaccharide nodulation factors (Sutton *et al.*, 1994). The pores formed by NodO in planar lipid bilayers are relatively large (>2 nm) and remain stably open for rather long, but are not voltage gated.

The *prsDE* genes for the TISS used by NodO appear to be conserved in all members of the *Rhizobiaceae* tested, even though these strains do not contain a *nodO* gene. The TISS, however, appears to be required for the secretion of several other Ca^2+^-binding proteins that are involved in the formation and the nitrogen-fixing capacity of nodules induced by *R. leguminosarum* bv. *viciae* (Finnie *et al.*, 1997). It also mediates the secretion of non-RTX glycanases PlyA and PlyB involved in the processing of *Rhizobium* exopolysaccharide (Finnie *et al.*, 1998).

### RTX proteins of unknown biological function

Initially, two partially homologous proteins, FrpA and FrpC, possessing the characteristic carboxy-proximal repetitions of the RTX nonapeptide motif, were discovered in *N. meningitidis*, a commensal of the human nasopharynx that occasionally causes invasive meningococcal disease (Thompson *et al.*, 1993a,b;). Biological activity of the Frp (Fe-regulated protein) proteins remains unknown. However, their secretion under iron-limited growth conditions, which mimic the condition in body fluids, and the elevated titers of antibodies against Frp proteins found in convalescent sera of patients from meningococcal disease, suggest a potential role of the FrpC-like proteins in meningococcal carriage or virulence (Osicka *et al.*, 2001).

Meningococci appear to carry a whole polymorphic family of *frpC*-like genes that code for proteins sharing large portions of identical sequence and varying in the number of C-terminal RTX repeat blocks and/or in insertions/deletions in the N-terminal portions. For example, the 122 kDa FrpA harbors 13 copies of nonapeptide repeats, while the 198 kDa FrpC has 43 copies. The N-terminal 293 residues of FrpA and the 407 N-terminal residues of FrpC do not exhibit any sequence homology, apparently due to the foreign DNA inserted at the 5′-end of the *frpA* gene (Thompson *et al.*, 1993b; Parkhill *et al.*, 2000; Tettelin *et al.*, 2000;).

The prototype FrpC protein of *N. meningitidis* FAM20 or MC58 strains is an 1829-residue-long protein with an amino-terminal portion of 876 residues and a carboxy-terminal RTX moiety of 953 residues. The N-terminal portion of FrpC (residues 1–414) does not exhibit significant homology to any known proteins. However, it binds with very high affinity the FrpD lipoprotein, which is expressed from a gene located immediately upstream of the *frpC* gene in a predicted iron-regulated *frpDC* operon (Prochazkova *et al.*, 2005). Recently, a unique ‘clip-and-link’ self-processing module (SPM) located between residues 414 and 657 of FrpC was characterized. It exhibits a high degree of sequence homology to segments of several RTX proteins of unknown function that are encoded in genomes of plant and animal pathogens (Osicka *et al.*, 2004). Upon binding of calcium ions, the SPM produces a novel type of autocatalytic cleavage of the peptide bond between residues Asp^414^ and Pro^415^ of FrpC. Moreover, the newly generated N-terminal fragment of FrpC can be covalently linked to another protein molecule by a novel type of Asp-Lys isopeptide bond, which forms between the carboxyl group of the C-terminal Asp^414^ residue of the thus generated fragment and the ɛ-amino group of an internal lysine of another protein molecule (Osicka *et al.*, 2004). This defines a novel class of autoprocessing RTX proteins of unknown biological function, as the same type of calcium-dependent processing and cross-linking activity was also demonstrated for the purified ApxIVA protein of *A. pleuropneumoniae* (Osicka *et al.*, 2004).

ApxIVA is specific to the species of *A. pleuropneumoniae* (Schaller *et al.*, 1999) and is genetically quite distant from other known RTX toxins. It is produced by *A. pleuropneumoniae* only during infections *in vivo*. When expressed as a recombinant protein in *E. coli*, it shows slight hemolytic activity and a distinct cohemolytic (CAMP) reaction. The *apxIV* determinant lacks the activator C gene and the type I secretion genes B and D, which are found in loci for the production of additional Apx toxins (ApxIA–ApxIIIA). However, upstream of *apxIVA*, an ORF can be found that is necessary for the hemolytic and CAMP activity of ApxIVA (Schaller *et al.*, 1999; Frey & Kuhnert, 2002;). ApxIVA was shown to be required for the full virulence of *A. pleuropneumoniae*, although its mechanism of action remains unclear (Liu *et al.*, 2009).

Recently, a system for single-step affinity chromatography purification of untagged recombinant proteins based on the SPM of *N. meningitidis* was developed (Sadilkova *et al.*, 2008). The N-terminus of the SPM is fused to a target protein of interest and the C-terminus to an affinity tag. Upon loading of cell lysate and binding of the fusion protein to an affinity matrix, contaminating proteins are washed away and site-specific cleavage of the Asp-Pro bond linking the target protein to the self-excising module is induced by the addition of calcium ions. This results in the release of the target protein with only a single aspartic acid residue added at the C-terminus, while the self-excising affinity module remains trapped on the affinity matrix (Sadilkova *et al.*, 2008).

## Identification of novel RTX proteins

Currently, almost 1000 prokaryotic genomes are fully sequenced and >2000 genome sequencing projects are in progress. We downloaded 840 fully sequenced bacterial genomes available in February 2009 and the putative RTX proteins were identified using three different methods: (1) pattern search; (2) Hidden Markov Model (HMM) search by hmmer 2.3.2 (Eddy, 1998); and (3) rps-blast (Marchler-Bauer *et al.*, 2002).

For the identification of RTX proteins by pattern search, we tested the Prosite pattern PS00330 (DX[L/I]XXXXGXDX[L/I]XGGXXXD) (Hulo *et al.*, 2006), the pattern proposed for the AC toxin GGXG(N/D)DX(L/I/F) (Bauche *et al.*, 2006), and a universal short pattern for the calcium-binding site GGXGXD. Matches to a database of all bacterial proteins were identified by the program preg, which is a part of the emboss package (Rice *et al.*, 2000). We analyzed >2.75 million sequences of a total length of 865 million amino acid residues. As expected, the longer the tested pattern was, the fewer the proteins that contained a positive hit. In total, 2598 hits to the Prosite pattern were found in 773 proteins. Shorter patterns were identified 5337 and 34 027 times, respectively, in 1210 and 20 963 proteins, respectively. To assess how many hits could be expected at random, we calculated an average amino acid composition of the bacterial protein database and generated a database of proteins of a random sequence, but with the same average composition. The average protein composition was calculated by pepstats and random sequences were generated by makeprotseq from the emboss package (Rice *et al.*, 2000). The overall size of the randomized database was equal to the size of the database containing all bacterial proteins. The randomized database was tested for the presence of the aforementioned patterns and randomization and testing was repeated 100 times. On average, only 1.6 hits to the Prosite pattern were found in the random database. Not surprisingly, the other two shorter patterns were more frequent, with 373 and 20 504 hits on average for GGXG(N/D)DX(L/I/F) and GGXGXD motifs, respectively. The specificity of shorter patterns in identifying RTX proteins could be improved by searching for multiple hits in a single protein. For the purpose of this review, we only considered proteins containing a hit to the Prosite pattern as being a putative RTX protein.

The alternative approach to identification of calcium-binding sites common to RTX proteins was to use the HMM-based model for hemolysin-type calcium-binding repeats, the PF00353 from Pfam database release 23.0 (Finn *et al.*, 2006), which is an 18-residue-long model of two calcium-binding sites. We used hmmer 2.3.2 (Eddy, 1998) for the identification of proteins containing these calcium-binding sites. One thousand and thirteen proteins exhibited significant homology to the PF00353 Pfam model. On average, only 0.1 hits were found in the above-described random database. Thus, this approach appeared to be highly specific and generated virtually no false-positive hits.

The last used approach was to predict RTX proteins with rps-blast (Marchler-Bauer *et al.*, 2002). We searched the bacterial protein database for homology to the 510-amino-acid-long COG2931, position-specific scoring matrix (PSSM) that represents RTX toxins and the related Ca^2+^-binding proteins. In contrast to Pfam models, there was no cutoff *e*-value defined for PSSM; therefore, we tested *e*-value cutoffs lower from 10, 1, 0.1 and 0.01. An *e*-value cutoff equal to or higher than 1 was not specific enough and >15 proteins were identified even in the random sequence database. Using an *e*-value of 0.1 as a cutoff, >400 proteins were identified in the bacterial database, compared with only 1.25 in the random sequence database. We therefore considered a cutoff of *e*-value equal to or <0.1 as being sufficiently stringent for the purpose of this review.

The combination of results from the three above-described methods revealed 1024 putative RTX proteins (Table S1) in 251 bacterial species. In bacterial genomes containing at least one putative RTX protein, components of the TISS were identified by hmmer (Eddy, 1998). Towards this aim, the following models from the TIGRFAM database version 8.0 (Haft *et al.*, 2003) were used: (1) TIGR01842, TIGR01846 and TIGR03375 for ABC transporters (PrtD, HlyB and LssB-like); (2) TIGR01843 for MFP (HlyD-like); and (3) TIGR01844 for OMP (TolC-like) (Table S2).

### Predicted RTX proteins

Bacteria harboring RTX protein genes were mostly *Gammaproteobacteria* (100 strains), *Alphaproteobacteria* (56 strains) and *Betaproteobacteria* (32 strains). Cyanobacteria were represented 25 times and RTX proteins appear, indeed, to be most abundant in cyanobacteria, with *Trichodesmium erythraeum* IMS101 bearing genes for 35 putative RTX proteins and up to 28 RTX proteins being encoded in the *Acaryochloris marina* MBIC11017 genome.

Surprisingly, putative RTX protein genes were also detected in seven Gram-positive bacteria genomes, with six *Actinobacteria* and one *Firmicutes* (*Streptococcus sanguinis* SK36), respectively. None of the *Actinobacteria* genomes, however, harbored any significant homologues of TISS components. Interestingly, the *S. sanguinis* SK36 genome contains an operon encoding an RTX protein, a HlyB-like protein and a HlyD-like protein. The TolC protein was not identified. A TolC homologue would, however, not be needed for Type I RTX secretion in a Gram-positive bacterium, which lacks the outer membrane. Whether the RTX protein and TISS components are really produced and functional in *S. sanguinis* remains to be determined.

**Fig. 6:**
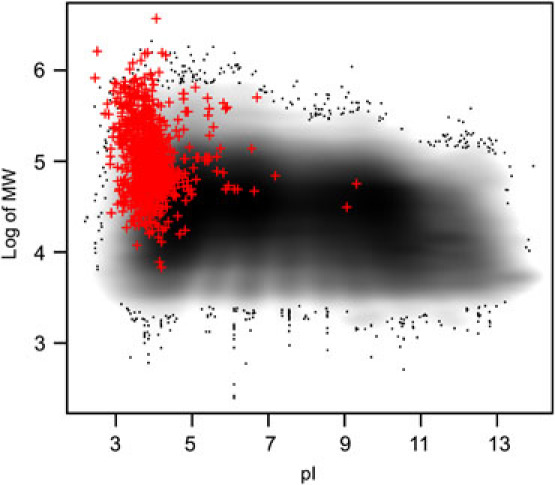
Comparison of size and pI distribution of the bulk of bacterial proteins and of the sum of characterized and putative RTX proteins. Molecular weight (MW) and pI was calculated for the bulk of bacterial proteins (about 2.75 mil., shown in black) and for the identified and predicted RTX proteins (1024 proteins, shown as red crosses) using the program pepstat, a part of the emboss package (Rice *et al.*, 2000). MW was log base 10 transformed (*y*-axis) and plotted against calculated pI (*x*-axis) in statistical package r version 2.9.0 (http://www.r-project.org/).

RTX proteins identified by the aforementioned approaches vary significantly in length and range from <100 residues (protein NP_437763 of *Sinorhizobium meliloti*) to 36 800 residues (protein YP_378930 of *Chlorobium chlorochromatii*). Ninety percent of the putative RTX proteins fit to the range of 300–5000 residues, with an average of about 1600 residues. With the exception of three proteins, all RTX proteins appear to be acidic, with a theoretical pI in the range from 3.2 to 4.9 for 90% of the putative RTX proteins (Fig. 6).

We further predicted a number of possible Ca^2+^-binding sites in the identified RTX proteins. The following consensus sequences were considered as potential binding sites for Ca^2+^: (1) GGXGX(D/N), (2) GXXGND, (3) GDXGXD, (4) GDAXXN, (5) GXGGXD, (6) GEAGDD and (7) GAGRVD, respectively. The highest number of almost 200 and 170 calcium-binding sites was predicted for the putative 4334-residue-long RTX protein of *Chlorobium limicola* and the 5107-residue-long protein from *Pseudomonas syringae* pv. *phaseolicola*, respectively.

Independent of the primary annotation, the prediction of functions of the putative RTX proteins was attempted. We used rps-blast ver 2.2.20 (Altschul *et al.*, 1997) to detect significant homologies of putative RTX proteins to PSSMs in the CDD database version 2.16 (Marchler-Bauer *et al.*, 2002), which contains almost 32 000 domain models from Pfam, COG, SMART and KOG databases. Based on detected homologies to the CDD database, we were able to assign putative functions to about 30% of the identified RTX proteins (Table S1). The largest group would consist of proteases (112) and adhesins (113), the latter falling into two major classes of cadherins (52) and vWA domain proteins, respectively (46). A further 20 lipases and 16 peroxidases were predicted.

About 20 enzymes were predicted to be involved in the degradation of saccharides and polysaccharides as glycosyl hydrolases, polysaccharide hydrolases and endoglucanases. About 20 putative RTX proteins might be involved in the degradation of DNA as endonucleases, nucleotidases and phosphodiesterases. A further four might act as phytases, one as a sulfatase, three as cyclophilin-like peptidylprolyl *cis*–*trans* isomerases and five as phosphatases, respectively.

Interestingly, three β-lactamases and one putative lysozyme were also predicted among the computationally detected putative RTX proteins. A further nine putative RTX proteins appear to exhibit weak homology to Hedgehog/Intein domain proteins, suggesting that they might undergo autoprocessing and splicing.

## Conclusions

**Fig. 7:**
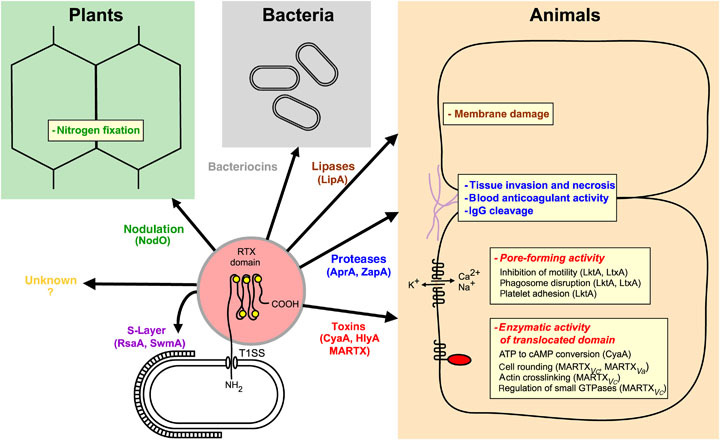
Use of a simple RTX building unit in generation of complex biological functionalities. By maintaining an unfolded state which allows a post-translational secretion from the calcium-depleted cytoplasm, and by promoting protein folding upon the binding of calcium ions (yellow balls) in the extracellular environment, the C-terminal assemblies of glycine- and aspartate-rich nonapeptide RTX repeat units first assist in the translocation of even very large RTX proteins. These transit across the entire Gram-negative bacterial cell envelope in a single step mediated by the dedicated the type I secretion machinery recognizing unprocessed C-terminal secretion signals. Proteins using this secretion pathway exhibit a very broad range of biological functions in colonizing diverse host environments. RTX proteins were found to exert activities like structural proteins involved in protective S-layer formation and motility of bacteria, in colonization of root nodules of plants by symbiotic bacteria, serving as bacteriocins on other bacteria, exerting hydrolase activities, or playing a prominent role as essential colonization and virulence factors of bacteria in animal hosts, respectively. Besides of a large group of pore-forming leukotoxins a particular sophistication of function is observed for the very large (thousands of residues long) RTX toxins consisting of multiple domains exhibiting enzymatic and cytotoxic activities (MARTX).

Being first recognized as a group of pore-forming bacterial leukotoxins two decades ago (Welch, 1991), the RTX family nowadays comprises a particularly broad range of exoproteins that play important roles in the colonization of various habitats and hosts by Gram-negative bacteria (Fig. 7). Bioinformatic mining of the growing database of bacterial genomes indicates that a large spectrum of biological and biochemical activities of RTX proteins still remains to be characterized. The unique and central overarching feature of RTX proteins is the presence of variable numbers of the calcium-binding glycine- and aspartate-rich C-terminal repeats. These separate the unprocessed C-terminal secretion signals from the specific bodies of individual proteins. By being intrinsically unstructured at the very low calcium concentrations found within bacterial cytoplasm, the repeats maintain an unfolded or a loosely folded state of the RTX protein that enables recognition of its secretion signal by a dedicated type I secretion ATPase assembly. This function of RTX repeats allows a single-step passage of extremely large proteins through the entire Gram-negative cell envelope. Binding of extracellular calcium ions to repeats emerging at the bacterial surface then ‘turns on’ structuration of the repeats, pulling the protein out of the cells and driving folding of the rest of the protein. Moreover, the RTX repeats themselves support an amazingly vast array of biological activities, be it a role in the formation of S-layers, bacterial adherence/motility or host–receptor interaction and membrane penetration of RTX proteins. Hence, through a simple variation of number and block arrangement of a small repeat unit, an elegant construction of sophisticated biological functions is achieved.

## Statement

Re-use of this article is permitted in accordance with the Terms and Conditions set out at: http://wileyonlinelibrary.com/onlineopen#OnlineOpen_Terms

## Supporting Information

**Table S1.** Predicted RTX proteins identified by bioinformatic screening of sequence databases.

**Table S2.** Bacterial genomes containing *rtx* genes, as identified by sequence database screening.
