# Correction to: RTX proteins: A highly diverse family secreted by a common mechanism

**DOI:** 10.1093/femsre/fuad024

**Published:** 2023-07-12

Irena Linhartová and others, RTX proteins: a highly diverse family secreted by a common mechanism, FEMS Microbiology Reviews, Volume 34, Issue 6, November 2010, Pages 1076–1112, https://doi.org/10.1111/j.1574-6976.2010.00231.x

Upon original publication, incorrect supplementary files were inadvertently included within this manuscript's Supplementary data section.

The correct supplementary files for this manuscript are provided with this correction notice; these details have been corrected only in this correction notice to preserve the published version of record.

## Supplementary Material

fuad024_Supplemental_Files

